# Adar-mediated A-to-I editing is required for embryonic patterning and innate immune response regulation in zebrafish

**DOI:** 10.1038/s41467-022-33260-6

**Published:** 2022-09-20

**Authors:** Katarzyna Niescierowicz, Leszek Pryszcz, Cristina Navarrete, Eugeniusz Tralle, Agata Sulej, Karim Abu Nahia, Marta Elżbieta Kasprzyk, Katarzyna Misztal, Abhishek Pateria, Adrianna Pakuła, Matthias Bochtler, Cecilia Winata

**Affiliations:** 1 International Institute of Molecular and Cell Biology in Warsaw, Warsaw, Poland; 2 Institute of Human Genetics, Polish Academy of Sciences, Poznan, Poland; 3 Polish Academy of Sciences, Institute of Biochemistry and Biophysics, Warsaw, Poland

**Keywords:** Transcriptomics, Gastrulation, RNA editing, Innate immunity

## Abstract

Adenosine deaminases (ADARs) catalyze the deamination of adenosine to inosine, also known as A-to-I editing, in RNA. Although A-to-I editing occurs widely across animals and is well studied, new biological roles are still being discovered. Here, we study the role of A-to-I editing in early zebrafish development. We demonstrate that Adar, the zebrafish orthologue of mammalian ADAR1, is essential for establishing the antero-posterior and dorso-ventral axes and patterning. Genome-wide editing discovery reveals pervasive editing in maternal and the earliest zygotic transcripts, the majority of which occurred in the 3’-UTR. Interestingly, transcripts implicated in gastrulation as well as dorso-ventral and antero-posterior patterning are found to contain multiple editing sites. Adar knockdown or overexpression affect gene expression by 12 hpf. Analysis of *adar*-/- zygotic mutants further reveals that the previously described role of Adar in mammals in regulating the innate immune response is conserved in zebrafish. Our study therefore establishes distinct maternal and zygotic functions of RNA editing by Adar in embryonic patterning along the zebrafish antero-posterior and dorso-ventral axes, and in the regulation of the innate immune response, respectively.

## Abstract (web summary)

Additional roles for A-to-I editing of RNA continue to be uncovered. Niescierowicz et al. report prevalent A-to-I editing in the zebrafish transcriptome, and the distinct maternal and zygotic functions of the editing enzyme Adar in embryonic patterning and in the regulation of innate immune response, respectively.

## Introduction

RNA editing is a phenomenon of post-transcriptional alteration of transcript primary sequence^1^. Its most common form is the A-to-I editing, in which adenosine (A) at the C6 position is deaminated, giving rise to an inosine (I)^2,3^. As inosine pairs like guanine with tRNAs, A-to-I editing has the potential to alter the coding capacity of mRNAs, in some cases with drastic biological consequences^4,5^. However, because of a prevalence of A-to-I editing in double-stranded RNA regions, most editing events do not affect the coding capacity of the genome.

A-to-I editing occurs widely in animals, from the earliest-diverging eumetazoan phyla to man^6,7^. In vertebrates^8^ and invertebrates^9^, A-to-I editing prevents autoimmunity that is triggered by endogenous dsRNA^10–12^. Another recurring theme of A-to-I editing is its role in the brain. In the mouse and zebrafish, editing of the GluR2 transcript is important for normal development of the nervous system^5,13,14^. In the fruit fly, perturbed A-to-I editing causes behavioral phenotypes^15^. In certain ant species, it determines caste-specific behavior^16^. In the squid nervous system, extensive A-to-I editing is more prevalent in the giant axon system compared to the cell body, indicating region-specific editing within a neuron cell^17,18^. In human and mouse, A-to-I editing contributes to germline integrity, by preventing the spread of Alu^19–21^ and SINE elements^22^, respectively. Higher editing prevalence in zebrafish testis and ovary compared to other organs^23^ may hint to a role of A-to-I editing for germline integrity in non-mammalian vertebrates. However, to our knowledge, there is no evidence for this role in invertebrates yet.

A-to-I editing is catalyzed by adenosine deaminases (ADARs)^3^. Most vertebrates have three paralogues that have arisen prior to vertebrate radiation, and can therefore be expected to have biochemically similar functions and substrate preferences^3,24^. Apart from a C-terminal deaminase domain, all ADARs have at least two, and in some cases three double-stranded DNA (dsDNA) binding domains. ADAR1 additionally has a Z-DNA-binding domain (ZBD) at the amino-terminal end of the protein that is missing from the other deaminases^25^. Among the ADAR paralogues, only ADAR1 and ADAR2 are active, whereas ADAR3 has an inactive catalytic domain and appears to fulfill its biological role in the absence of catalytic activity^26,27^.

In mammals, ADAR1 and ADAR2 are widely expressed, whereas ADAR3 is only expressed at low levels in the brain^3^, particularly in the amygdala and hypothalamus^26^. The majority of A-to-I editing is performed by ADAR1 and prevents dsRNA mediated autoimmunity^11^. ADAR1/Adar1 occurs as two isoforms, known as p110 and p150^28^. Both share the deaminase domains, the dsRNA-binding domains, and one Z DNA-binding domain. Additionally, p150 possesses a second Z DNA-binding domain at the N-terminal end^29^. Mice with a homozygous knockout of either both isoforms or only the p150 isoform cannot complete embryonic development. They die between embryonic day 11.5 and 12.5 from failed erythropoiesis and fetal liver disintegration^30,31^, presumably due to stress in these cells^32^. At the molecular level, Adar-mediated RNA editing was shown to regulate innate immune response by maintaining basal levels of A-to-I editing on self-RNA to prevent it from being recognized as viral RNA^33,34^. In contrast to *Adar1* null mice, *Adar2* mutant mice can complete embryonic development, but die subsequently from seizures, within 3 weeks of birth^5^. Remarkably, this phenotype depends on a single editing event in the GluA2 AMPA receptor transcript. Even though multiple transcripts in the brain are edited^4^, a point mutation in the GluA2 transcript suffices to suppress the Adar2 null phenotype. In humans, ADAR2 is associated with epilepsy, neurodegeneration, and autism^35^.

In zebrafish, almost all data about A-to-I editing are descriptive. In contrast to most other vertebrates, which have three ADAR paralogues, zebrafish have four, due to a duplication of the ADAR2 ortholog into *adarb1a* and *adarb1b*. In the following, we use the official zebrafish nomenclature: *adar* for adar1, *adarb1a* for *adar2a*, *adarb1b* for *adar2b*, and *adarb2* for *adar3*. Transcripts for *adar*, and to a lesser extent *adarb1b*, are highly abundant during the first few hours of development. Transcripts of the other adenosine deaminase genes are scarce or absent during the first few hours. Although they are eventually expressed later on, the transcript levels remain lower than those of *adar* and *adarb1b* throughout development. Sequencing data suggests that editing in transcripts from repetitive genomic elements is pervasive in the first few hours of development, before the maternal to zygotic transition, and much less pronounced in later developmental stages^23^. Editing in coding regions of genes sets in only later, roughly one day after fertilization. In adult organs, *adar* was most highly expressed in testis and heart, whereas *adarb1a* was highly expressed in heart and brain. Overall A-to-I editing was most pervasive in testes and ovaries^23^. In contrast to the detailed information about the occurrence of editing, very little is known about functional consequences. It is known from earlier work that editing of GluR2 is conserved in zebrafish and is essential for normal development of the nervous system and cranial neural crest cells^14^. However, it is unclear how conserved the role of A-to-I editing is otherwise, particularly for the early stages of development that differ greatly between mammals and zebrafish.

Here, we explore the role of A-to-I editing in early zebrafish embryos, focusing on the most highly expressed *adar*. Knockdown and overexpression experiments revealed that maternal *adar* is essential for zebrafish development, particularly during the earliest steps of anteroposterior and dorsoventral patterning, and that this function is dependent on an intact deaminase domain. Transcriptome analysis uncovered prevalent A-to-I RNA editing during early embryogenesis, which affects transcripts known to play a role in gastrulation as well as dorsoventral and anteroposterior patterning. *adar* knockout experiments further demonstrated a role of Adar in the regulation of innate immune pathway—a function which is conserved with that in mammals.

## Results

### Adar and Adarb1b are expressed during zebrafish development

**Fig. 1:**
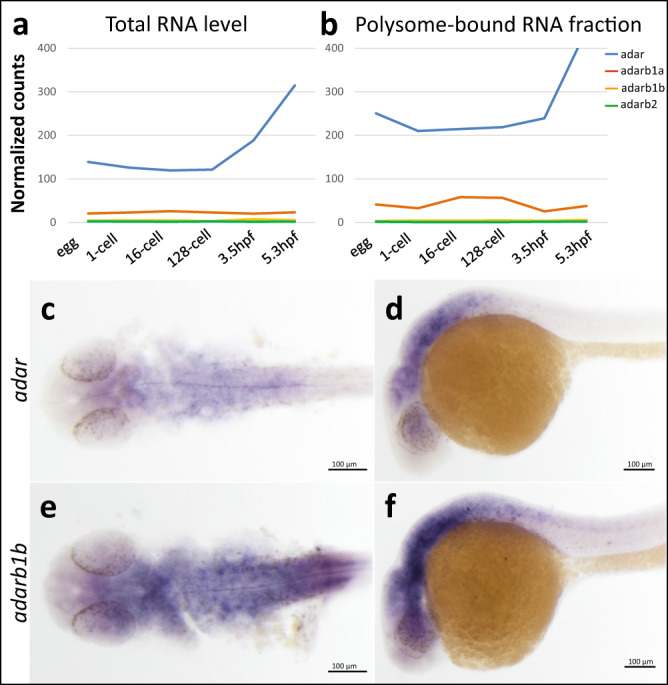
Spatiotemporal expression pattern of Adar family genes in zebrafish. **a** Expression of zebrafish *adar* family based on transcriptome profiling of developing embryos (RNA-seq)^53^. Expression levels are plotted for developmental stages: egg, 1 cell, 16 cells, 128 cells, 3.5 hpf, and 5.3 hpf. Levels of **a** total RNA and **b** polysome-associated RNA of four zebrafish *adar* paralogs are given. Whole mount in situ hybridization shows expression pattern of *adar* (**c**, **d**) and *adarb1b* (**e**, **f**) in 24 hpf zebrafish embryos. Each experiment was performed three times on embryos from three independent mating pairs with similar results. Source data are provided as a Source data file.

To determine whether A-to-I editing activity exists during embryonic development, we revisited our transcriptome data^36^ to check whether the enzymes responsible for A-to-I editing were expressed. In agreement with other recently published data^23^, we detected transcripts of at least two deaminase paralogs, *adar* and *adarb1b*, from egg to 5.3 hpf (Fig. 1a). Transcripts of these two paralogs were present both maternally as well as zygotically, with *adar* being more abundant (more than 4-fold compared to *adarb1b* at each developmental stage). Moreover, we found that transcripts of both paralogs were consistently associated with polysome, starting from the egg stage up to 5.3 hpf (Fig. 1b). This finding suggests that these transcripts are constantly undergoing translation at developmental stages preceding and after the activation of zygotic genome^36^. The observation that both paralogs were already expressed, and their transcripts associated with polysomes at egg stage suggests that RNA editing events occur prior to fertilization and may be crucial for early development. Interestingly, a substantial increase in *adar* expression occurs after the mid-blastula transition (MBT), suggesting that the role of this gene extends beyond the period of transcriptional silence in early embryogenesis (Fig. 1a). At larval stage, the expression of both gene paralogs was not spatially restricted although more abundant in the nervous system of the developing embryo as shown by whole mount in situ hybridization of *adar* (Fig. 1c, d) and *adarb1b* (Fig. 1e, f) in 24 hpf zebrafish embryos.

### Adar editing activity is required for embryonic patterning

**Fig. 2:**
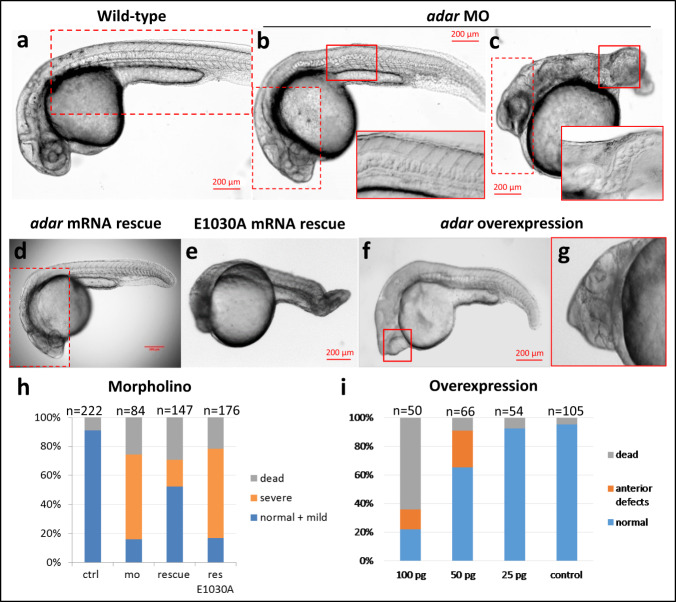
Phenotypic defects at 24 hpf caused by *adar* knockdown and overexpression. **a** Wild-type (**b**, **c**) Adar MO-injected embryos develop abnormal phenotype in the posterior part with disturbed body axis, shortened tail and crooked, disorganized notochord. **d** MO phenotype can be fully rescued with wild-type mRNA injection. **e** Mutant *adar* mRNA E1030A with inactivated editing domain could not rescue the malformed phenotype. **f**, **g** Phenotype defects caused by *adar* mRNA overexpression. The anterior defects, including cyclopia and head malformations are mRNA dose dependent. Inset marked by red boxes denotes overlaid identical image taken at different focal plane. Observation was performed on embryos from four independent adult pairs with similar results. **h**, **i** Injection statistics of Adar MO, rescue, and mRNA overexpression. Source data are provided in the Source data file.

To investigate whether Adar plays a biological role in early zebrafish development, we used morpholino oligonucleotides (MO) to knockdown Adar, the highest expressed among all zebrafish ADAR paralogs (Fig. 1a). Adar MO-injected embryos developed a range of phenotypes which is initially evident at gastrulation and subsequently observed to affect posterior body axis by 24 hpf (Fig. 2a–c, h). The most severe phenotypes manifested in a lack of almost all posterior structures and crooked body axis (Fig. 2c). In addition, the notochord in morphant embryos was disorganized, with unevenly shaped and distributed vacuoles instead of the neat “stack of coins” arrangement in wild-type (Fig. 2b, c). This abnormal MO phenotype was dose dependent and could be rescued with the wild-type *adar* mRNA, in which an increased percentage of embryos appeared normal or exhibited mild phenotype with proper body axis organization and tail length (Fig. 2d, h; Supplementary Fig. 1). On the other hand, knockdown with up to 2 ng of MO against Adarb1b, which was expressed at a lower level in the early embryo, did not result in any observable phenotype (Supplementary Fig. 1). To verify whether the biological role of Adar depends on its RNA editing activity, we generated *adar* mRNA E1030A designed after a similar construct in mammals^37^, in which the deaminase domain was mutated (Supplementary Fig. 1). The E1030A mRNA was unable to rescue MO-induced phenotype in developing embryos, resulting in comparable number of abnormally developed embryos with severe posterior axis defects to that of MO-injected larvae (Fig. 2e, h). This suggests that a functional deaminase domain, catalyzing the A-to-I editing in dsRNA, is essential for early embryonic development. These results strongly suggest that the A-to-I editing activity of Adar is necessary for the specification of early embryonic axes.

Whereas Adar MO affected the development of the posterior part of the body, *adar* mRNA overexpression caused significant abnormalities of the anterior part, which include anomalous eye development, most often manifested in cyclopia, deformed cranium, and reduced or absent brain compartment, particularly at the anteriormost region (Fig. 2f, g, i). These defects were most prominently observed with 50 pg injection of mRNA. The effect of *adar* mRNA overexpression was dose dependent with a 64% mortality for the 100 pg of injected mRNA, 9% for 50 pg and 7% for 25 pg (compared to wt which had 5% mortality). Overexpression of the E1030A mRNA did not lead to an abnormal phenotype, further confirming that the function of Adar is deaminase-dependent (Supplementary Fig. 1B). Collectively, our results show that Adar plays a key role in the earliest steps of embryonic patterning.

**Fig. 3:**
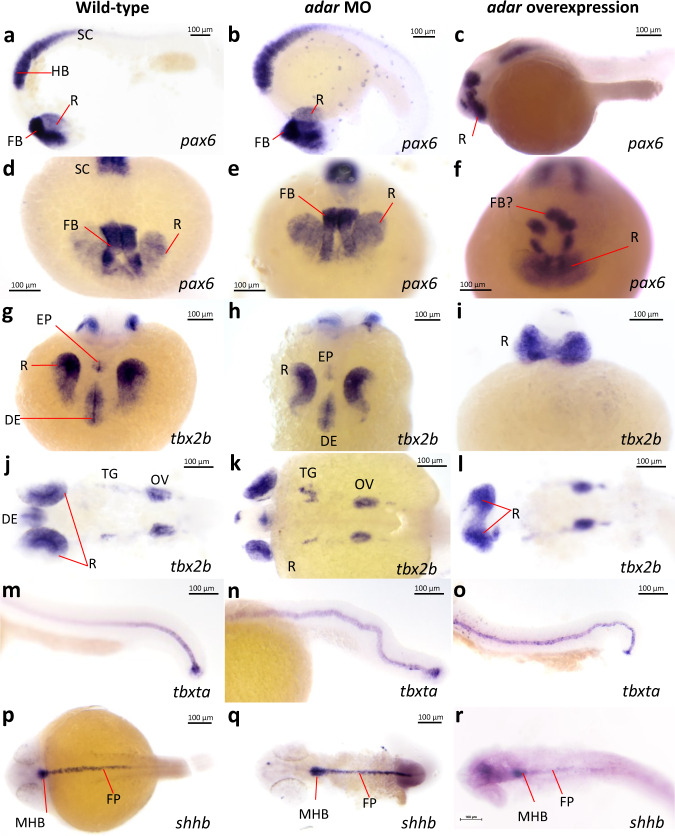
Embryonic patterning defects due to Adar loss- or gain-of-function. Whole mount in situ hybridization detection of marker genes in Adar morpholino knockdown and *adar* mRNA overexpression. **a**–**f** *pax6* expression demarcates anterior brain structures including dorsal retina (R), forebrain (FB) as well as hindbrain (HB) and anterior spinal cord. **g**–**l** *tbx2b* expression marking diencephalon (DE), R, epiphysis (EP), trigeminal ganglion (TG), and otic vesicle (OV). **m**–**o** expression of *tbxta* marks the notochord. **p**–**r** *shhb* expression indicates floor plate (FP) and midbrain-hindbrain boundary (MHB). The experiment was performed three times on embryos from three different mating pairs with similar results.

In order to characterize the observed morphological defects of Adar loss- and gain- of function in more detail, we assessed the expression of several marker genes indicating various embryonic structures. The most prominent phenotype of Adar disruption is that of the development of structures along the anteroposterior axis. In order to characterize the effect of Adar loss- or gain-of function on the development of anterior embryonic structures, we utilized the expression of *pax6* and *tbx2b* to collectively indicate the forebrain, midbrain, and hindbrain, anterior spinal cord, dorsal retina, and otic vesicle (Fig. 3a, d, g, j)^38,39^. In *adar* morphants where morphological defects were predominantly observed in the posterior region, these anterior structures were preserved and appeared similar to wild-type in terms of their size and organization (Fig. 3b, e, h, k). On the contrary, anteriormost brain regions and retina were indistinguishable in embryos overexpressing *adar* (Fig. 3c, f, i, l). In particular, *pax6* expression shows that the forebrain expression domain were unrecognizable in *adar*-overexpressing embryos (Fig. 3c, f). Moreover, *tbx2b* expression domains of diencephalon, left and right retina, and the epiphysis appeared as a fused region, whereas the more posterior expression domains of the trigeminal ganglion and otic vesicles appeared less affected although hypomorphic compared to control (Fig. 3i, l). While the eye field is derived from the anterior neural plate and therefore arose as a consequence of anteroposterior patterning^40^, its failure to split in the midline (cyclopia) is a hallmark of a defect in the convergent-extension movement during gastrulation which is dependent on the establishment of the dorsoventral axis^41,42^. The observed phenotype of *adar* overexpression therefore suggests that proper establishment of these two body axes was affected.

To examine the defects in posterior structures, we used as reporter genes *tbxta* and *shhb* which mark the notochord and floor plate neurons of the spinal cord, respectively (Fig. 3m, p). Similar to the anterior markers, *shha* and *tbxta* were expressed in the case of both *adar* loss- or gain-of-function, suggesting that the neural and midline cell identities were preserved (Fig. 3n, o, q). However, the body axis including the notochord and spinal cord were crooked in *adar* morphants as well as in *adar* overexpressing embryos albeit to a lesser extent in the latter (Fig. 3n, o). Importantly, loss- or gain-of-function of *adar* do not appear to affect the specification of cell identity as evidenced by the preserved expression of *pax6, tbx2b, shha,* and *tbxta*. Rather, a more pronounced effect is seen on the gross anteroposterior and dorsoventral patterning of the embryo. Collectively, these observations suggest that Adar-mediated A-to-I editing may be involved in the patterning along the two early embryonic axes of the zebrafish.

### Prevalent A-to-I editing in maternal and early zygotic transcripts

**Fig. 4:**
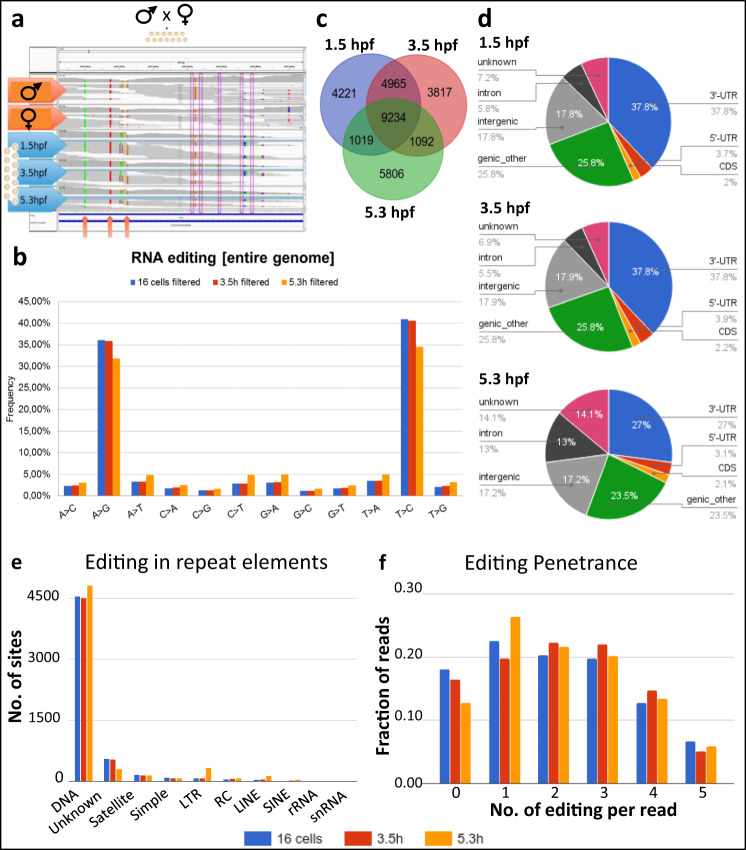
A-to-I RNA editing sites in early embryonic development. **a** Schematics of A-to-I RNA editing discovery through sequencing of a parent-offspring trio. The genome sequence of the parents are used as a reference set to distinguish between polymorphisms and editing. **b** Mismatches between RNA and DNA sequencing data. As RNA libraries were not strand selective, mismatches were read as their complement (i.e., T->C instead of A->G, or C->T as G->A) in roughly half of all cases. **c** Overlap of editing sites at different time points. The 1.5 and 3.5 hpf samples were more similar to each other than to the 5.3 hpf sample, probably because of replacement of maternal by zygotic transcripts at the MZT. **d** Association of editing sites with genomic features. A large fraction of RNA editing is classified as “genic_other” due to overlap between introns/exons/UTRs from multiple transcripts. **e** Number of editing sites in transcripts stemming from different classes of repeat elements. **f** Number of editing events in individual reads encompassing 10 potential editing sites. The majority of individual reads contained 1–3 RNA editing sites, and never more than five editing sites. Source data: Supplementary Data 1–3, Source data file.

The effects of Adar disruption on embryonic patterning led us to ask to what extent A-to-I editing occurs in early embryogenesis, and whether transcripts of genes involved in this process were specifically affected. To profile global A-to-I RNA editing in zebrafish we sequenced a trio of wild-type sample of both parental genomes and the transcriptome of their offspring at three developmental stages: 1.5 hpf (16 cells, pre-MBT), 3.5 hpf (high, MBT) and 5.3 hpf (50% epiboly, post-MBT) (Fig. 4a). Comparison of zygotic transcriptomes with the genomic sequence of parents allowed us to pinpoint RNA editing events by identifying mismatches between the genomic and transcriptomic reads. As Inosine (I) is structurally similar to a G due to the presence of the 6-oxo group, reverse transcriptase incorporates a C in the corresponding position during RNA-seq library synthesis; thus, in the original transcript strand, a G is inferred. Therefore an A-to-I editing event can be identified as an A–G mismatch between the parental genome and the corresponding embryonic transcript. Since the RNA-seq was unstranded, the same applies as well to a T–C mismatch. Strikingly, our analyses revealed a disproportionate enrichment of A–G and T–C mismatches compared to other possible base mismatches which could occur stochastically at all three developmental stages (Fig. 4b). This strongly suggests the presence of A-to-I RNA editing in maternally deposited as well as in zygotic transcripts. Altogether, we identified 44,007 RNA editing sites: 11,374 of which were shared by all three samples, 6352 between 1.5 hpf and 3.5 hpf, 1393 between 1.5 hpf and 5.3 hpf, and 1,564 between 3.5 hpf and 5.3 hpf. Apart from this, 6117, 5686 and 11,521 were specific to 1.5 hpf, 3.5 hpf and 5.3 hpf, respectively (Fig. 4c, Supplementary Data 1). These stage-specific editing events indicate differential patterns of RNA editing throughout early embryogenesis and suggest that this process may play a role in embryogenesis. Interestingly, merely 2% of RNA editing sites occurred within coding sequences (CDS), while the majority (38% in 1.5 hpf and 3.5 hpf and 27% in 5.3 hpf) occurred in 3’-UTR regions (Fig. 4d, Supplementary Data 1). A large fraction of editing was assigned as ‘genic_other’ due to overlap between intron/exon/UTRs from multiple transcripts. Finally, ~17% of editing events in each stage were detected in ‘intergenic sequence’, but this may be due to unannotated transcripts. The low abundance of A-to-I editing present in the coding regions (CDS), none of which resulting in missense mutations, ruled out the possibility that editing functions through expanding the coding repertoire of expressed transcripts by altering amino acid composition. We also observed plenty of editing occurring in DNA repeat regions with a notable increase in editing frequency in retrotransposons (LTR, LINE, and SINE) in 5.3 hpf compared to earlier developmental stages (Fig. 4e, Supplementary Data 2).

To assess the penetrance of A-to-I editing on individual RNA molecules, we calculated the occurrence of editing events in RNA-seq reads. Based on alignment of 75 bp reads, we observed a substantial number of reads with no editing at all for a given region (Fig. 4f). Interestingly, the number of RNA editing sites in a single read never exceeds 50% of the total number of editing events identified in the given transcript region (Fig. 4f, Supplementary Data 3). This suggests that A-to-I editing rarely occurs at all possible editing sites in a given RNA molecule, although sequencing with much longer reads is required to precisely determine the true penetrance. We then ask whether RNA editing events were associated with translation efficiency and/or RNA stability. Using our previously published polysome profiling dataset to determine translation rate^36^, we found that transcripts undergoing more active translation tend to be less edited than those not associated with polysome (Supplementary Fig. 2). Interestingly, this is true only for editing events detected at post-MBT stages (3.5 hpf, *r* = −0.053, *p* = 3.79e−11;and 5.3 hpf, *r* = −0.071, *p* = 2.36e−22), but not for pre-MBT (1.5 hpf, *r* = 0.002, *p* = 0.78). With regards to RNA stability, no obvious difference was observed in expression levels between low (1–9 sites) and high (more than 10 sites) edited transcripts when 1.5 hpf and 3.5 hpf stages were compared. However, highly edited transcripts undergo much lower expression change between 1.5 hpf and 5.3 hpf (Supplementary Fig. 3).

**Fig. 5:**
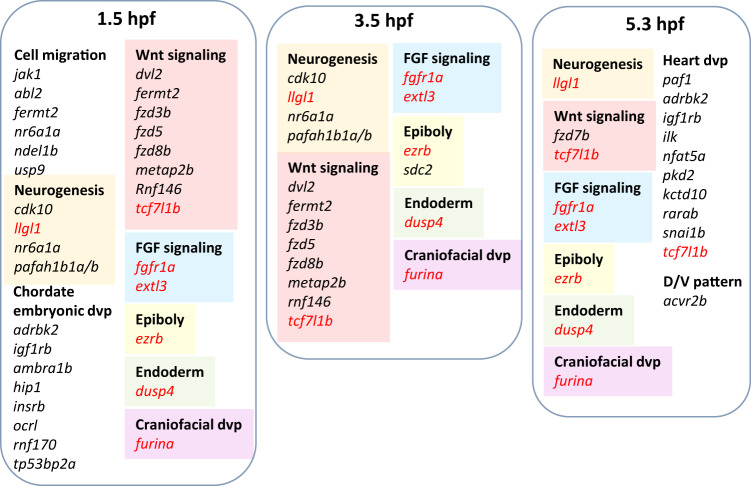
Selected transcripts of developmental signaling pathway genes implicated in dorsoventral and/or anteroposterior patterning containing two or more RNA editing sites detected at their 3′-UTR region. Gene names in red denotes those that are commonly edited at all three stages. Source data: Supplementary Data 4.

We sought to identify which genes were subject to A-to-I editing at each developmental stage. We identified 639, 634, and 562 genes having at least two editing sites at any position within their transcript at 1.5 hpf, 3.5 hpf, and 5.3 hpf respectively (Supplementary Data 4). Among these, 9, 9, and 10 genes had editing sites occurring in both 3’UTR and coding sequence at each respective stage. Interestingly, although no developmentally relevant GO terms were found to be significantly enriched among edited genes (Supplementary Data 5), we found several key genes known to be implicated in anteroposterior and dorsoventral patterning (Fig. 5). Of note, members of the Wnt signaling pathway were found to be edited. Several Frizzled receptors of Wnt signaling, *fzd3b*, *fzd5*, and *fzd8b* were edited at both maternal stages, while *fzd7b* were edited at 5.3 hpf. The Wnt downstream effector *tcf7l1b* was found to be consistently edited at all three stages. We also found two members of the FGF signaling pathway, *fgfr1a* and *extl3*, consistently edited at all three stages observed. Interestingly, *fgfr1a* is one of the most highly edited transcripts, containing 99 edited sites in both its coding sequence and 3’-UTR. Mutation of FGFR1 in humans is associated with holoprosencephaly^43^ which is reminiscent of the observed *adar* overexpression phenotype. Other genes which were consistently edited throughout the three stages include *furina* which plays a role in craniofacial development^44^ and two genes involved in gastrulation, *dusp4* and *ezrb*^45,46^. That transcripts of these genes were found to be edited throughout maternal and early zygotic stages suggests the role of A-to-I editing in regulating multiple aspects of anteroposterior and dorsoventral patterning.

### Adar loss of function affects dorsoventral patterning

To uncover the role of Adar at the molecular level, we performed transcriptome sequencing of wild-type, Adar MO knockdown (KD) and Adar overexpressing (OE) embryos at 128 cells (before ZGA), 5.3 hpf (after ZGA), and 12 hpf (when embryonic patterning is established). We hypothesized that alterations in the level of *adar* transcripts may evoke consequential differences in mRNA editing and/or changes in the expression of potential target genes. To this end, we performed comparative transcriptome analysis of wt vs. OE and wt vs. KD in each developmental stage. Surprisingly, no substantial changes in global A-to-I editing levels were found in KD and OE conditions at 128-cell and 5.3 hpf stages (Supplementary Fig. 4A). Moreover, during these two stages, samples clustered according to developmental stage (Supplementary Fig. 4B) and no substantial changes were observed in global gene expression profile (*p* < 0.05; Supplementary Fig. 4C). Only 539 sites have a slightly elevated editing level in OE than in KD in both developmental stages (Supplementary Data 6).

**Fig. 6:**
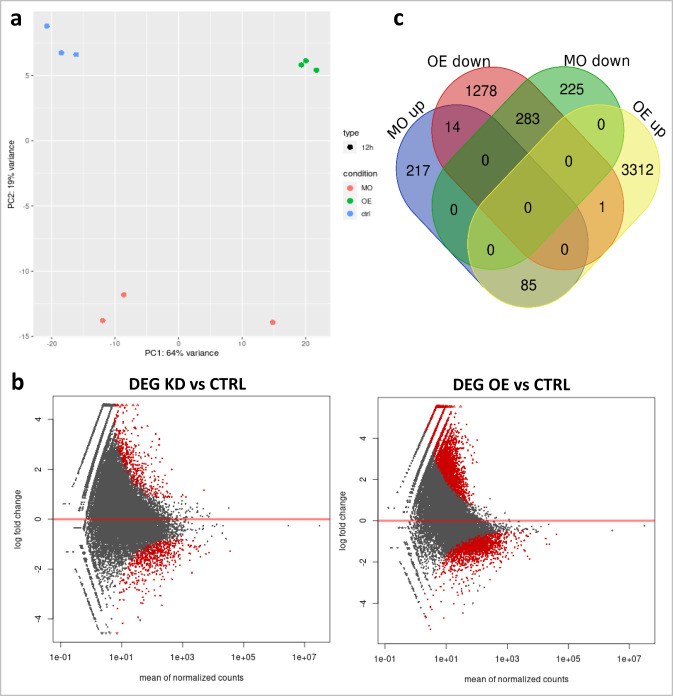
Differentially expressed genes at 12 hpf in Adar KD and OE. **a** Principal component analysis of control, Adar knockdown and overexpression samples based on their transcriptome profile. **b** Number of genes differentially expressed in each condition and their overlap. **c** Differential expression analysis of Adar KD and OE compared to control. Genes with significant change in expression (*p* < 0.05) in red. Sequencing read counts were normalized with DESeq2 standard method. Statistical significance is determined by the Wald test corrected for multiple testing using the Benjamini and Hochberg method using DESeq2. Source data: Source data file, Supplementary Data 7.

In contrast, at 12 hpf, a more noticeable change in both global editing pattern and gene expression profile was observed between control and Adar gain- or loss-of-function at 12 hpf (Fig. 6a; Supplementary Fig. 5). Unlike at earlier stages, samples clustered according to conditions rather than developmental stage (Fig. 6b). Interestingly, in all three replicates, Adar KD caused a modest but more noticeable change in editing frequency compared to the earlier stages (Supplementary Fig. 5). Moreover, at 12 hpf, we observed 827 and 5054 genes differentially expressed in Adar KD and Adar OE respectively, compared to control (*p* < 0.05; Supplementary Data 7). GO analysis (Supplementary Data 8) revealed that Adar KD generally caused the upregulation of genes regulating epiboly (*nanog, cacnb4b*), gastrulation (*bcl2l10, mylipa*), and ectoderm development (*pou5f3, cdh1*), while concurrently causing downregulation of those implicated in convergent-extension (*wnt11f2, creb1a, ppp1cb*) and mesoderm development (*tbx16, hes6, her7, mcdh2, myf5, msgn1*). On the other hand, Adar OE resulted in the upregulation of genes involved in the development of mesodermal structures (*myf6, bves, tcf21, tbx5a, apln, mef2aa*), while downregulating genes regulating epiboly (*chuk, epcam, mapkapk2a, cldne*), dorsoventral pattern formation (*sox11b, dusp6, ved, vox, bambia, acvr1ba, ctnnb2*), and brain development. Interestingly, out of the 383 genes common between the two conditions, 283 commonly downregulated genes in both Adar KD and OE included genes known for their role in anterior-posterior as well as dorsoventral patterning (Fig. 6c; Supplementary Data 7), such as *cdx4*^47^, *szl* and *ved* required for DV patterning^48,49^, and several *hox* genes^50^. This suggests that both Adar KD and OE may act on factors with downstream consequence of suppression of these axis-regulating genes.

As Adar gain- and loss-of-function resulted in almost opposite defects in anterior-posterior and dorsoventral patterning, we compared the transcriptome from the two conditions and found 1218 differentially expressed genes (Supplementary Data 7). Genes regulating the development of mesodermal structures (*bmpr2a, emilin3a, msgn1, gata1a, meis1b, etv2, pdgfra*) as well as *hox* family genes (*hoxa4a/9b, hoxb3a/5a, hoxc1a/3a/6a, hoxd4a*) were upregulated in OE compared to KD (Supplementary Data 8). On the contrary, genes regulating epiboly and gastrulation (*chuk, epcam, mapkapk2a, snai1a*), convergent-extension (*dsc2l, gpc4, tdgf1, ptpra, prmt1*), and dorsal-ventral patterning (*sox11b, ctnnb1, ppp4cb, tll1*) were downregulated in OE compared to KD (Supplementary Data 8). These observations agree with the phenotypes resulting from Adar disruption which affected structures along the anteroposterior and dorsoventral axes, where an excess of Adar affected dorsal and anterior structures while Adar deficiency affected more ventral and posterior structures. Taken together, the consequences of Adar disruption at the molecular level could be observed by 12 hpf, which affected the expression of genes involved in the development of various embryonic structures. This suggests that, although no significant changes were observable in global editing levels and transcriptome up to 5.3 hpf, Adar is necessary during the crucial period of embryonic patterning, between gastrulation and 12 hpf.

### Zygotic Adar is not essential for early embryogenesis

**Fig. 7:**
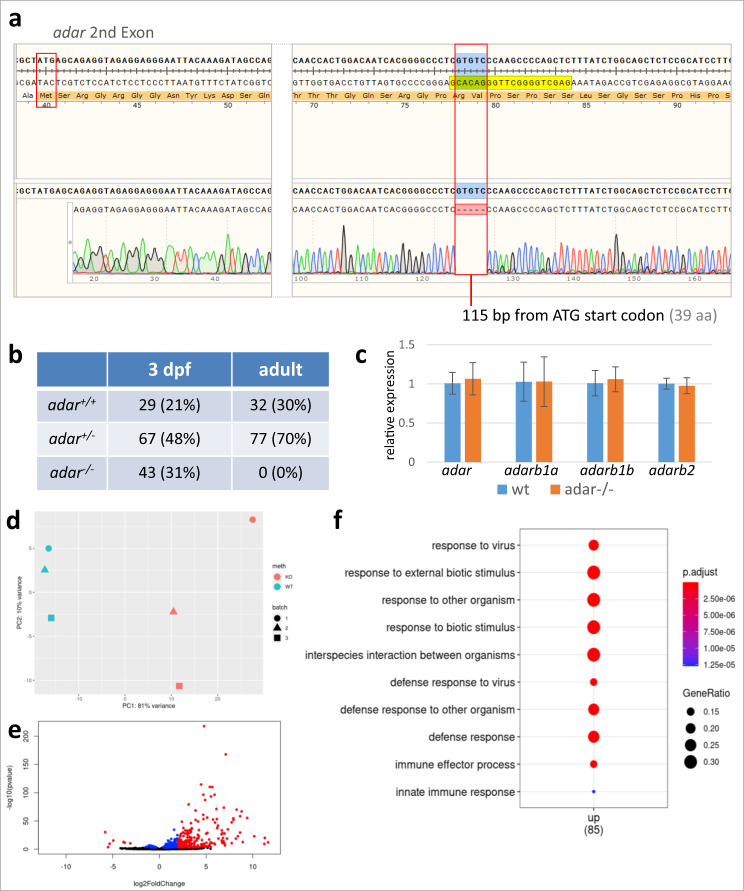
Zygotic *adar−/*− mutant exhibit increased innate immune response. **a** *adar−/*− mutant generated by CRISPR/Cas9 had a 5-bp deletion within the second exon, resulting in a premature stop codon at 115 bp position. **b** Different genotype ratios at different time points suggesting that *adar−/*− homozygotes die between 3 dpf and adult stage. **c** transcript levels of *adar* and its paralogs as measured by qRT-PCR at 3 dpf show the lack of genetic compensation effect in homozygous mutant individuals as compared to wild type. Values shown are average of 5 individuals in each WT and *adar−/*− groups. Data are presented as mean values ± SD. **d** principal component analysis of wild-type and adar*−/*− based on their transcriptome profile at 7 dpf. **e** differential expression analysis revealed a number of significantly upregulated and downregulated genes, which were enriched in functions associated with innate immune response. **f** Statistical significance for differentially expressed genes is determined by the Wald test corrected for multiple testing using the Benjamini and Hochberg method using DESeq2. Functional enrichment analysis was performed using DAVID which employs Fisher’s Exact test with Benjamini, Bonferroni corrections. Source data: Source data file, Supplementary Data 11, 12.

We then asked whether zygotic Adar activity is required for later events of embryogenesis. To this end, we created a zebrafish *adar* mutant line (*adar−/*−) using the CRISPR/Cas9-based gene knockout method. A 5 bp deletion was introduced into the second exon of the *adar* gene, resulting in a premature stop codon and polypeptide consisting of 4% (39 aa) of the full length, functional Adar protein (917 aa) (Fig. 7a). To generate homozygous *adar* mutants we incrossed F1 heterozygous individuals and observed the F2 offspring. Surprisingly, no developmental phenotypes were observed in any of the offspring. We then raised these F2 individuals and genotyped them at ~3 months post-fertilization by fin clipping. Surprisingly, we found 76% heterozygous and 24% wild-type fish among the 200 screened samples. None of the adult F2 individuals were homozygous for the *adar−/*− mutant allele (Fig. 7b). This suggests that the homozygous *adar−/*− knockout animals are not viable despite their lack of developmental abnormalities. To determine the fate of the homozygous *adar−/*− mutants, we genotyped F2 individuals (~300 individuals) at 3 dpf. Interestingly, the distribution of homo-, heterozygous and wild-type individuals was in agreement with the Mendelian ratio (Fig. 7b), indicating that mortality of the homozygous *adar−/*− individuals occurred later than 3 dpf and before adulthood. In order to pinpoint the timing of mortality, we followed the development of homozygous individuals until the point of death. The *adar−/*− larvae did not demonstrate any observable morphological abnormalities when compared to heterozygotes or wild-type individuals. Moreover, the swimbladder of *adar−/*− individuals inflated normally at the same time as that of its wild-type siblings at 5 dpf. However, significant mortality was observed between second- and third weeks post fertilization. Nearly 100% of *adar−*/− mutants died within this period, with only one surviving until the third month post fertilization, with substantial growth impairment. To eliminate the possibility of genetic compensation^51^ causing an attenuation of the *adar−/*− phenotype, we analyzed mRNA levels of *adar* as well as three other *adar* family members: *adarb1a, adarb1b* and *adarb2*, in wild-type and *adar−/*− homozygotes. No significant changes in the mRNA levels of *adar*, *adarb1a, adarb1b and adarb2* mRNAs were observed in *adar−/*− individuals when compared to wild-type at 3 dpf (Fig. 7c). Our findings therefore suggest that the lack of phenotypic alterations in *adar−/*− larvae, when compared to *adar* KD (MO-injected) was not due to genetic compensation. It is possible that the late mortality of zygotic *adar* KO stems from the presence of maternally deposited *adar* mRNAs and/or proteins from heterozygous mothers, which might be sufficient to drive early developmental processes in the first few days post fertilization. Altogether, these observations suggest that zygotic Adar is not required for early embryogenesis although its function is still essential for life later on.

### Loss of zygotic *adar* cause widespread innate immune response

Adar1 loss of function in mammals is characterized by an induction of innate immune response^33^. In order to test whether this function is conserved in zebrafish, we performed transcriptome analysis on homozygous *adar−/*− individuals close to the time in which mortality starts to be observed. To this end, we isolated RNA from homozygous *adar−/*− and its wild-type siblings at 7 dpf and performed RNA-seq. Our analyses revealed that in homozygous *adar−/*− individuals, 193 and 7 genes were significantly (*p* val < 0.05) upregulated and downregulated more than fourfold compared to wild-type, respectively (Fig. 7d, Supplementary Data 11). Functional enrichment analysis of overexpressed genes suggest highly significant enrichment of genes related to the innate immune response (Fig. 7e). Notably, genes encoding components of the MDA5-mediated interferon response known to be implicated in mammalian Adar deficiency were found to be highly upregulated in homozygous *adar−/*− individuals. These include the zebrafish MDA5 orthologue *ifih1*, as well as the effectors of this pathway *irf3* and *irf7* ^52^. Another group of interferon-stimulated factors, the IFIT proteins, are known to be transcriptionally induced as a response to viral infection^53,54^. Strikingly, seven members of this protein family were highly upregulated, including *ifit8, ifit9, ifit10, ifit12, ifit14, ifit15*, and *ifit16*. In addition to interferon-response genes, those encoding members of the ubiquitin-proteasome degradation pathway associated with interferon response were found to be upregulated, including *psmb8a* and *psmb13a* which are zebrafish orthologues of the interferon inducible subunits β5i and β2i respectively^55,56^, as well as several genes encoding ubiquitin modifiers and ligases (*isg15, usp18, uba7*, and *CABZ01058333.1* encoding E3 ubiquitin-protein ligase rnf213-alpha-like). Moreover, apoptosis-related genes were also upregulated, including the caspases *xaf1*, *casp21* and *casp23*.

We then searched for specific features of zygotic editing and sought to identify the changes in editing profile caused by the loss of Adar. Interestingly, similar to that observed during embryonic stages, A-to-G conversion were the most enriched among other possible conversions (Supplementary Fig. 6). The editing events at 7 dpf were also prevalent at the 3′-UTR, suggesting that the preference for editing in this region is likely a general feature of Adar activity. Intriguingly, despite the prominent innate immune upregulation observed, we could not see significant alterations in editing pattern between wild-type and *adar−/*− mutants (Supplementary Fig. 6). Moreover, genes containing editing sites were also not enriched for any particular function, including that of innate immunity (Supplementary Fig. 6, Supplementary Data 12).

In summary, the loss of zygotic Adar function in zebrafish induced a widespread innate immune response, proteasome-mediated degradation and apoptosis by 7 dpf, which likely led to death within the second to third weeks post-fertilization. Importantly, the activation of the innate immune response as a result of A-to-I editing deficiency in zebrafish is consistent with the MDA5-dependent Type I interferon response observed in mammalian ADAR deficiency^8,34^. Our results therefore establish conservation of the zygotic function of Adar between fish and mammals.

## Discussion

### Editing primarily in the 3′-UTRs of coding transcripts

Sequencing data in this work indicate that a very small percentage of A-to-I editing in zebrafish takes place in the protein-coding regions, with the majority occurring in the 3′-UTRs of coding transcripts. Editing primarily in the 3′-UTRs and outside the coding regions of transcripts is expected based on similar patterns observed in mammals^57^. It is also consistent with a recent data for zebrafish^23^ that editing within the protein-coding regions of transcripts sets in after 24 h. However, we also note that the previous study applied very conservative criteria for editing (to keep the false positive rate low) and called editing sites in embryonic transcripts based on a catalog of editing sites for adult brain samples^23^. Therefore, this study may systematically miss editing sites that are unique for the embryo and not found in the brain. Prior studies have shown that in mammals ADAR2 is more associated with editing in coding sequences, whereas ADAR1 edits more promiscuously, either in transcripts from repetitive genomic elements or, in case of coding transcripts, in the 3′-UTR regions^27^. The data in this work confirm that the latter is conserved in zebrafish. Secondary structure elements located in 3′-UTR in zebrafish maternal mRNAs have been reported to be involved in translational regulation of mRNAs of the Nodal family by RNA binding proteins^58^. Therefore, it is reasonable to expect that RNA editing may participate in such regulation or that similar but distinct secondary structure elements are preferred by Adar in vivo, providing a combinatorial regulatory code for fine-tuning translation and/or degradation rates. The knowledge of secondary structures adopted by maternal mRNAs in vivo is therefore becoming highly necessary to better understand the mechanism of gene expression regulation.

### No effect of Adar knockdown on transcript editing

The delayed onset of embryonic transcription and reliance on maternal transcript deprives early embryos of the opportunity to regulate gene expression transcriptionally^36^. This limitation, together with the very high *adar* transcript levels in early zebrafish embryos suggested to us that Adar activity could be used to control transcript and thus protein levels prior to the MZT, as an alternative to transcriptional control. Surprisingly, however, Adar knockdown or overexpression had very limited effect on global A-to-I editing until 5.3 hpf, i.e., until well after the MZT at 3.5 hpf ^59^. Phenotypic data and the editing data for the 12 hpf time point rule out a technical problem with knockout. It is possible (but hard to confirm in the absence of good antibodies) that a sufficient pool of maternal Adar protein is deposited in the oocyte and dominates editing in the early stages (up to 5.3 hpf), but eventually gets exhausted to reveal editing differences in the knockdown (at 12 hpf).

Intriguingly, the same lack of editing alterations were also observed in Adar null mutants at the zygotic stage, which suggests that A-to-I editing is robust and that other Adar paralogs may perform a complementary role to maintain a certain level of editing although still insufficient to ensure normal function of the system. Alternatively, editing changes as a consequence of Adar knockdown could be more subtle than anticipated. For instance, editing pattern alterations could occur within a particular transcript molecule, or different proportions of transcripts for particular genes could be edited as a result of Adar disruption. Unfortunately, the short-read sequencing method used in our study is not able to detect these differences. Long read sequencing methods such as the Oxford Nanopore promise an opportunity to further explore this in the near future.

### Different pools of Adar may explain the discrepancy between knockdown and knockout phenotypes

While the Adar KD by morpholino induced severe patterning defects, *adar−/−* mutant individuals are viable without obvious embryonic defect. Successful rescue of the knockdown phenotype with a morpholino-resistant transcript makes it unlikely that off-target effects account for the difference in editing. Genetic compensation was also ruled out in mutants. Thus, the difference in phenotype between Adar KD and mutant could be attributed to distinction of maternal and zygotic function of Adar. Instead, we suspect that maternally deposited Adar transcripts account for the difference. Due to the eventual lethality of the *adar* knockout, the *adar−/−* embryos were from *adar(+/−)* heterozygous parents. Therefore, there is a pool of maternally derived *adar* transcripts in the *adar(−/−)* embryos. By contrast, translation of the maternally derived *adar* transcripts is blocked in the morpholino experiments. We suspect that a pool of Adar proteins from embryonic translation of maternal Adar transcript spares the knockout embryo the anteroposterior and dorsoventral defects that we see in the knockdown embryos. Eventually, however, this Adar pool is probably turned over, so that defects, now purely reflecting the embryonic genotype, become apparent. The detailed causes for this late phenotype are not yet clear. Adar null fish live vastly longer (until the second or third week) than would be expected based on death of the *adar1* null mice from hematopoietic defects (at embryonic day 12 in the mouse, equivalent to 36 hpf in zebrafish^60^). Therefore, similar causes of death are unlikely. Instead, the late phenotype points to other, still unexplored roles of A-to-I editing in late larval development.

### Distinction between maternal and zygotic function of Adar

Strikingly, knockdown and overexpression of *adar* resulted in opposite phenotypes, with the former abolishing posterior-ventral structures including the differentiation of notochord, while the latter affecting anterior-dorsal ones resulting in cyclopia. We also found that this phenotype is dependent on an intact RNA editing domain of Adar. These phenotypes are reminiscent of those caused by disruptions to several dorsoventral and anteroposterior axis determinants. Two signaling pathways are known to be responsible for this process: Wnt and FGF. Loss of Wnt signaling is known to cause a severe antero-dorsalized phenotype where embryos possess large heads and truncated tails, while its overactivation results in the opposite phenotype of posteriorized embryos lacking eyes^49,61,62^. Similarly, loss of FGF signaling also causes truncation of the posterior body due to the lack of posterior mesoderm structures^63–65^. Intriguingly, we observed that several Wnt and FGF pathway components were edited throughout the maternal and zygotic stages, suggesting a link between Adar function and the observed editing events. To our knowledge, a role of A-to-I editing in embryonic patterning is has not been seen before in any vertebrate. Clearly, the very different early embryology of zebrafish compared to mammals creates opportunities for new biological outcomes of A-to-I editing that deserve further study.

While maternal Adar is required for embryonic patterning, analysis of zygotic Adar mutants revealed a distinct function in regulating innate immune response. Homozygous *adar−/−* individuals developed normally up to free-feeding larval stage, revealing that zygotic Adar function is dispensable for embryogenesis. However they start to decline after the first week post-fertilization with a significant upregulation of innate immune response accompanied by activation of proteasomal degradation pathways and widespread apoptosis in the embryo. The stimulation of innate immune response was also observed in mammalian Adar mutants^33,34^, as well as liver apoptosis and hematopoiesis failure^30,31^. Although the effects of Adar dysfunction do not necessarily manifest in the damage of the same tissues as in mammals, the underlying molecular mechanism of Adar regulation of innate immune response seems to be conserved in fish.

Taken together, our study establishes a role of A-to-I editing by maternal Adar in regulating embryonic patterning, and a conserved role of zygotic Adar in keeping the innate immune response in check.

## Methods

### Zebrafish

The study complies with the guidelines on animal experimentation according to the EU Directive 2010/63/EU on the protection of animals used for scientific purposes. Zebrafish of wild-type (AB strain) and CRISPR/Cas9-generated *adar* mutant lines were maintained in the zebrafish core facility of the International Institute of Molecular and Cell Biology in Warsaw (IIMCB) (License no. PL14656251) according to standard procedures. Embryos were raised in egg water at 28 °C and staged according to standard morphological criteria^66^.

### MO injection, rescue and *adar* overexpression

Adar and Adarb1b knockdown was performed using a translation-blocking antisense morpholino oligonucleotides (MO) with the sequences 5′-TCCCTCCTCTACCTCTGCTCATAGC-3′ and 5′-TCCATGATGGTCAAACGTCTCGACT-3′ (Gene Tools, USA). For each embryo, 1–3 ng of MO was injected at the 1-cell stage. For overexpression experiments, the *adar* cDNA sequence was PCR-amplified using the primer pair 5′-CCTGTCTTTGATACTGTCGTG-3′ and 5′-TCCCGAAGCCACAGATTCAC-3′ and cloned into p-GEMT vector (Promega, USA). For the rescue experiment, wild-type and E1030A mutant *adar* cDNAs containing a 5 bp mismatch at the MO recognition site were PCR-amplified using the forward primer 5′-CCTAGCTAATACGACTCACTATAGGCGGAACATGAGTAGAGGAAGAGGAGGGAATTAC-3′ with a T7 promoter sequence overhang for in vitro transcription and reverse primer 5′-TCAAGCTATGCATCCAACGCG-3′. Capped mRNAs for rescue and overexpression were synthesized using the mMessage mMachine T7 Kit (Ambion, USA). Overexpression was done using 25, 50, or 100 pg of mRNA. Rescue experiments were performed using 1 ng of MO and 25 pg of wild-type or E1030A *adar* mRNA. Results were obtained from four different experiments on embryos from random pairs.

### Disruption of the deaminase domain in *adar* mRNA

Zebrafish Adar glutamate 1030 (E1030) in a highly conserved region of the protein (TVNDCHA**E**IISRRGFIRFLYSELM) was identified as equivalent to human ADAR1 glutamate 912 (E912), a residue known to be indispensable for catalytic activity of the deaminase domain. To encode the E1030A point mutation in the zebrafish *adar* clone, we used the Q5® Site-Directed Mutagenesis Kit (NEB) for mutagenic primer-directed replication of both plasmid strands. The first step was an exponential amplification using primers and a master mix formulation of Q5 Hot Start High-Fidelity DNA Polymerase. Oligonucleotides used for glutamate substitution with alanine and site-directed mutagenesis are as follows: zb_adar_E1030A_F 5′-GCAGCTATCATCTCCAGAAGAGGC-3′ and zb_adar_E1030A_R 5′-ATAGCTGCATGGCAGTCATTTACAG-3′. The second step involved an incubation with an enzyme mix containing a kinase, a ligase and *Dpn*I allowing for rapid circularization of the PCR product and removal of the template DNA. The last step was a high-efficiency transformation into chemically competent TOP10 cells. Selected clones were sent for sequencing and positive ones were linearized and used for in vitro *adar* E1030A mRNA synthesis.

### qPCR for genetic compensation analysis

Total RNA was extracted from genotyped *adar* homozygous mutant and wild-type 3 dpf larvae, using TRIZOL LS (Thermo Fisher Scientific, USA) according to the manufacturer’s protocol, followed by DNAse I (Life Technologies, USA) treatment. Superscript IV reverse transcriptase (Life Technologies, USA) was used to obtain cDNA. Relative mRNA expression was quantified using FastStart SYBR Green master mix on the LightCycler 96 instrument (Roche Life Science, USA) with specific primer pairs (Supplementary Data 9). Genetic compensation analysis was performed on 5 individuals from each WT and *adar* homozygous mutant groups.

### Sequencing and data analysis

For editing discovery, parental DNA was extracted from one individual male and female from tail-fin clip, fragmented by sonication with Covaris and sequencing library were synthesized with TruSeq Chip Library preparation kit (Illumina, USA) according to the manufacturer’s protocol. RNA from their offspring was also isolated at 16-cell, 3.5 hpf, and 5.3 hpf stages. For each time-point, at least 20 embryos were pooled for RNA extraction and processed for Truseq RNA Library preparation (Illumina, USA). To assess the effect of Adar KD and OE, uninjected wild-type and embryos injected with 1 ng of adar MO or 50 pg of adar mRNA were kept until the desired developmental stage: 128-cell, 50% epiboly, and 12 hpf. Two replicates of 20 pooled embryos from the first two time points and three replicates for the 12 hpf time point were isolated for RNA extraction. The RNA for the 12 hpf sample was collected in a second round of experiments, from offspring of a separate mating pair. Total RNA was extracted using TRIzol LS (Thermo Fisher Scientific, USA) and cleaned up on the Qiagen Rneasy Mini column (Qiagen, USA). Quality control of extracted RNA was performed using the 2200 TapeStation system from Agilent Technologies (USA). To avoid the bias caused by cytoplasmic polyadenylation during early embryonic stages, polyA affinity was not used for mRNA enrichment. Instead, total RNA was rRNA-depleted using Ribo-Zero Magnetic Gold Kit (Human, Mouse, Rat; Epicenter). cDNA synthesis for Next-Generation Sequencing (NGS) was performed by SMARTer Stranded RNA-seq kit (Clontech Laboratories, USA) as recommended by the manufacturer. Paired-end sequencing (2 × 75 bp reads) was performed with NextSeq 500 (Illumina, USA). Reads were aligned to the zebrafish genome assembly GRCz10 using STAR v2.7.7a^67^ and samtools v1.11^68^. On average, more than 80% of total sequencing reads were uniquely mapped (Supplementary Data 10). Expression quantification was performed using HTSeq v0.11.2^69^. Differential expression was performed using DESeq2 (R v3.6.3)^70^. HTseq reads from KD and OP samples were compared to the control samples at corresponding time points. Multiple testing was done by applying the Benjamini-Hochberg correction as implemented in DESeq2 with adjusted p-values <0.05 called as statistically significant. For the analysis of adar zygotic function, total RNA from homozygous *adar*−/− mutant and their wild-type siblings were isolated at 7 dpf from three replicates of 3 individuals each. Total RNA was subjected to mRNA enrichment using NEBNext Poly(A) mRNA Magnetic Isolation Module (E7490, NEB) followed by TruSeq Stranded Total RNA library preparation kit (Illumina, USA) according to the manufacturer’s protocol with minor adjustments. In brief, 1 μg of total RNA was used to enrich for polyadenylated RNA fraction. Obtained RNA was fragmented for 8 min in 94 °C to target final library size around 310 bp followed by first and second strand cDNA synthesis. To enrich for adaptor-ligated molecules samples were pre-amplified with 4 cycles and left aside on ice. In order to determine an optimal number of PCR cycles and limit the possibility of generating PCR duplicates and artifacts, a subsequent qPCR reaction on previously pre-amplified samples was performed. Final libraries were validated in terms of quality and quantity by Quantus fluorometer (Promega) and Tapestation 2020 High Sensitivity D1000 assay (Agilent Technologies). Pair-end sequencing (2 × 100 bp) was performed on Illumina NovaSeq 6000 using NovaSeq 6000 S1 Reagent Kit v 1.5 (200 cycles) (Illumina) to target a depth of 25 million reads per sample. Sequencing data were demultiplexed, and preprocessed with a Snakemake pipeline. Reads were subjected to quality-control using fastp^71^. At the 5′- and 3′-ends, 15 and 3 nucleotides were removed respectively based on atypical base composition according to fastqc/multiqc^72^. The truncated reads were then aligned to the zebrafish genome assembly GRCz11 using STAR v2.7.7a^67^, paired (option fixmate), sorted, and (again) deduplicated (option markdup -r) using samtools^68^. Based on the zebrafish genome annotation (z11.105). the reads were then translated to feature counts using HTSeq (option -s reverse)^69^. From the feature counts onwards, analysis was done in R using custom scripts. Differential expression analysis was carried out in DESeq2^70^. Significantly overexpressed and underexpressed genes (−2 > log2FC > 2; padj < 0.05) were annotated using bioMart^73^, and subjected to GO analysis using clusterProfiler^74^. The analysis is available under https://github.com/mbochtler/zebrafish_ADAR/7dpf/analysis_overexpression.

### RNA editing discovery

Putative RNA editing sites based on DNA- and RNA-seq input were detected using REDiscover (https://github.com/lpryszcz/REDiscover). The script automatically eliminates low quality and duplicate reads. It utilizes samtools mpileup to generate a text file from input bam files. Options -q 15 -Q 20, were used, i.e., a minimal mapping quality of 15 and a minimal base call quality of 20 were required. Sites that were not homozygous between female and male samples were excluded from the analysis. Alternative alleles were only called when they were present in at least 20% of RNA reads. For simplicity, sites with more than one alternative allele were excluded from the analysis. Between 57,605 and 150,495 putative edited sites were detected per sample (Supplementary Data 1). These sites were then filtered in a post-processing step that required a minimum coverage of 10 reads in both the DNA-Seq and RNA-Seq data.

For the 7dpf samples, the A > I editing discovery pipeline had to be modified, due to the lack of reference DNA sequencing, and the use of fish from a different genetic background. Extensive SNP data for zebrafish are available only for Zv9 (https://snpfisher.nichd.nih.gov/snpfisher/tracks.html)^75^. Hence, editing discovery for the 7dpf samples was done with reference to Zv9. After primer removal and quality control as for read quantification, reads were mapped using STAR^67^, and analyzed using REDiscover. Next, all sites with SNPs in the FLI, TL, and WK backgrounds were excluded, and a proximity filter was applied. The filter exploits the occurrence of editing sites in clusters. Edits of any chemical type were only accepted when at least 4 of neighboring 9 edits were of the same type. After this filtering step, calls for edits other than A > G (in DNA, corresponding to A > I in RNA), C > T (in DNA, corresponding to C > U in RNA), and unexplained G > A edits were reduced to a few percent at most (Supplementary Fig. 6A). The full analysis is available under https://github.com/mbochtler/zebrafish_ADAR/7dpf/analysis_A_to_I_editing.

### Whole-mount in situ hybridization

Whole-mount in situ hybridization was performed on 24 hpf wild-type, *adar* morpholino-injected and *adar* mRNA-injected embryos. Results were obtained from at least three different experiments on embryos from random pairs. Dechorionated embryos were fixed in 4% PFA for 2 h at room temperature, washed in PBS/Tween (PBT) and then sequentially dehydrated in methanol. After 100% methanol overnight incubation at −20 °C, the embryos were rehydrated in serial methanol dilutions, washed in PBT, digested with 10 µg/mL Proteinase K (Roche) for 5 min and refixed in 4% PFA for 20 min at room temperature. Then, they were washed in PBT and incubated in hybridization buffer (Hyb; 5× SSC, 50 µg/ml heparin, 0.1% Tween 20, 500 µg/mL tRNA, 50% formamide) in a water bath at 68 °C. After 4 h, the embryos were incubated in hybridization buffer containing 1 µg/mL DIG-labeled RNA probe overnight at 68 °C. Thereafter we performed several post hybridization washes: serial Hyb/2x SSC dilutions for 10 min each at 68 °C, 2 × 10 min 2× SSC at 68 °C, 2 × 15 min 0.2× SSC at 70 °C and serial 0.2× SSC/PBT dilutions at room temperature. The embryos were incubated in blocking solution (2 mg/mL BSA, 2% sheep serum and 1× PBT) at room temperature for 3–4 h, after which they were incubated in anti-DIG antibody (Roche, 1:5000) in the dark at 4 °C overnight. They were washed several times in PBT and incubated in alkaline phosphatase buffer (NTMT; 0.1 M Tris-HCl pH 9.5, 50 mM MgCl_2_, 0.1 M NaCl, 0.1% Tween 20) 3 times for 5 min each. We then stained the embryos by incubating them with NBT/BCIP solution (Roche) at room temperature. When the desired staining intensity was reached, the reaction was stopped by washing the embryos in PBT and fixing them in 4% PFA. Pictures were taken using Nikon SMZ25 stereomicroscope.

The *adar*, *adarb1a*, *adarb1b*, *gsc*, *foxa2*, *shhb*, and *tbxta* clones were amplified from a cDNA template using specific primers containing a T7 promoter sequence overhang at the reverse primer (Supplementary Data 9), and the corresponding riboprobe was synthesized using the DIG-RNA labeling kit (Roche) according to the manufacturer’s instructions. Clones for *pax6* and *tbx2b* were a kind gift from Vladimir Korzh.

### CRISPR/Cas9-mediated *adar* knockout in zebrafish

sgRNAs targeting the *adar* gene were designed using the CCTop on-line tool^76^. Three potential sequences were chosen based on their vicinity to the start codon as well as lack of predicted off-target exonic sites. Each sgRNA was tested by co-injecting it with Cas9 mRNA into 1-cell stage embryos. At 24 hpf genomic DNA was extracted from single embryos using the HOTSHOT method^77^. Genomic DNA was then used as a template for HRM analysis using sets of primers specific to the targeted region of the gene. DNA from single uninjected embryos was used as negative control. Based on the highest percentage of edited embryos, sgRNA_3 (5′-GAGCTGGGGCTTGGGACACG-3′) was deemed the most efficient and used for subsequent knockout line generation. In order to establish the mutant line, embryos of wild-type (AB) line fish at 1-cell stage were injected with 40 pg sgRNA_3 and 400 pg Cas9 mRNA each. The embryos were then raised to adulthood and outcrossed with wild-type AB fish. DNA from embryos resulting from this spawning was extracted and analyzed using the HRM approach as described before to screen for germline transmission^78^. Outcross of edit-carrying F0 individuals with wild type resulted in 25% of heterozygotes (F1). Offspring of incrossed F1 heterozygotes were raised to 3 months post fertilization and subsequently genotyped to identify mutant homozygotes (*adar*−/−). Oligonucleotides used for genotyping are as follows: forward 5′-CTTTCAGAAAGGGACAGCCTCAG-3′ and reverse 5′-GCGGAGAGCTGCCAGATAAAG-3′. Similarly, 3 dpf larvae from F2 generation were genotyped according to a published protocol^79^ and sequenced using the abovementioned pair of oligonucleotides.

### Statistics and reproducibility

No statistical method was used to predetermine sample size. Group size was based on our previous experience. Most of the experiments were repeated at least twice. The exact number of biological replicates used for statistical analysis is stated for each experiment. No data was excluded from the analysis. Unless otherwise stated, all sequencing experiments included biological replicates and were analyzed using the same bioinformatics pipelines, wherever possible keeping analyses parameters constant to ensure reproducibility. Statistical analysis applied for each experiment is mentioned in each corresponding methods section and figure legends. All attempts at replication were successful. RNA editing discovery was performed with a single trio of parental genomic sequencing and offspring transcriptomes at three different time points. The reliability of the initial A-to-I editing discovery is dependent on the parental genomic sequence. Therefore, sequencing was performed in a single trio of samples (male and female parental genome and offspring transcriptome) as the cost of sequencing additional sets are prohibitive. The offspring transcriptome was obtained as a result of pooling multiple embryos (20 individuals) which therefore dilutes out any biological variance. Samples were allocated to experimental groups according to their biological attributes which include developmental stage, genotype, or treatment type. These attributes defines each group clearly enough such that randomization is not necessary. Investigators were not blinded to group allocation during data collection. The analyses of compared groups were performed with the same pipelines and parameters and thus not subject to observer bias.

### Reporting summary

Further information on research design is available in the Nature Research Reporting Summary linked to this article.

## Supplementary information

Supplementary Information

Description of Additional Supplementary Files

Supplementary Data 1-12

Reporting Summary

## Data Availability

All sequencing data generated in this study have been deposited in the GEO database under accession number GSE182714. Processed data are provided as Supplementary Data. Raw data upon which figures are constructed are provided as a Supplementary Data. Source data are provided with this paper.

## Source data

Source Data

## Code availability

The code used to analyze data within this paper have been made publically available on GitHub at the following locations: https://github.com/mbochtler/zebrafish_ADAR/7dpf/analysis_overexpression, https://github.com/lpryszcz/REDiscover, and https://github.com/mbochtler/zebrafish_ADAR/7dpf/analysis_A_to_I_editing.
