# Supplementary material for: Adar-mediated A-to-I editing is required for embryonic patterning and innate immune response regulation in zebrafish
Source: Nat Commun. 2022 Sep 20;13:5520. doi: 10.1038/s41467-022-33260-6 (PMC9489775; doi:10.1038/s41467-022-33260-6)
Supplement: Supplementary file 1 — Supplementary Information [file 41467_2022_33260_MOESM1_ESM.pdf]

Supplementary Information

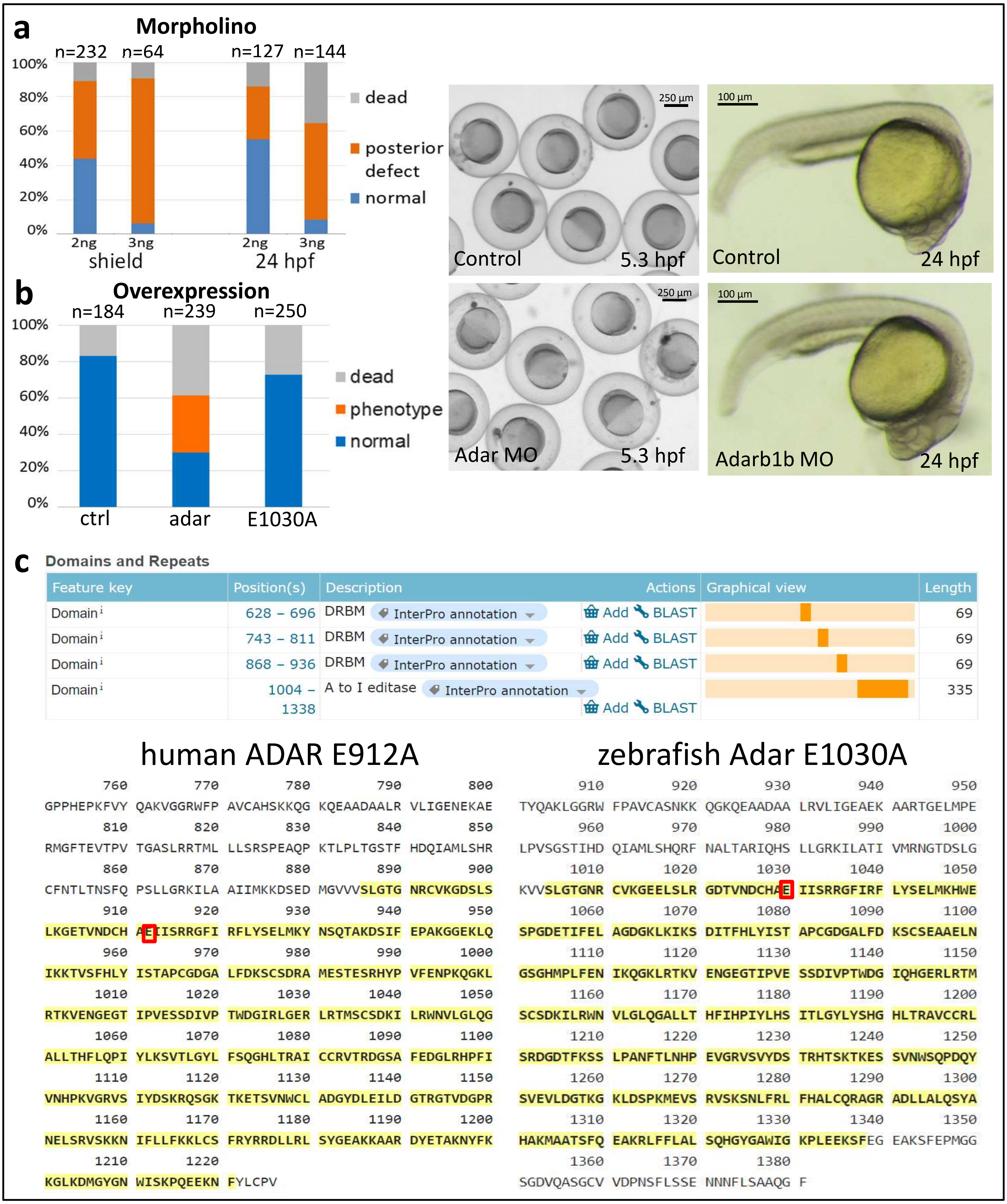

Supplementary Figure 1. Adar and Adarb1b knockdown experiments. (A) Dose-dependent effect of Adar MO was observed starting from 5.3 hpf, while Adarb1b MO did not cause any observable phenotype. (B) Overexpression of *adar* E1030A mRNA did not induce patterning defects observed in wild-type *adar* mRNA overexpression. Source data is provided as Source data file. The experiment was performed on embryos from four independent adult pairs with similar results. (C) Design of *adar* mutant mRNA E1030A with point mutation abolishing the activity of the deaminase domain.

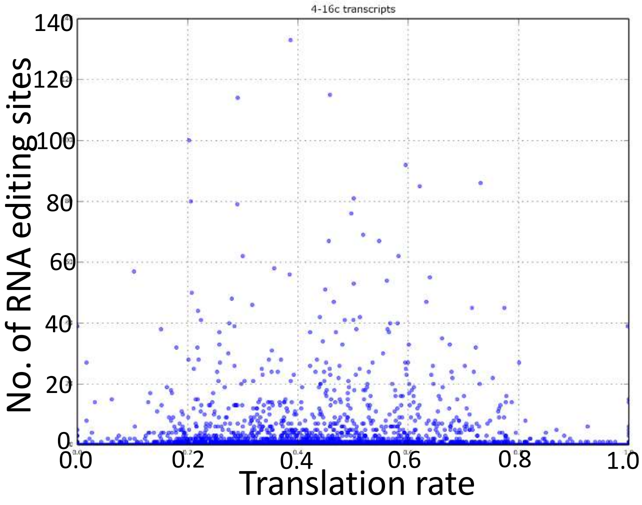

1.5 hpf

Pearson: r=0.002 p=0.78

Spearman: r=0.016 p=0.04

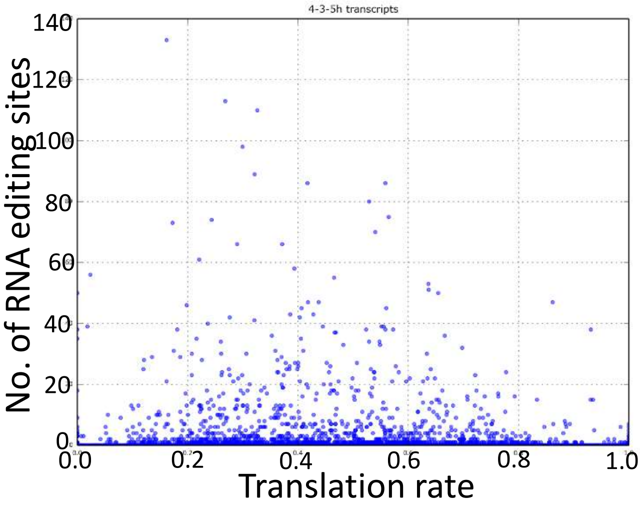

3.5hpf

Pearson: r=-0.053 p=3.79e-11

Spearman: r=-0.077 p=2.36e-22

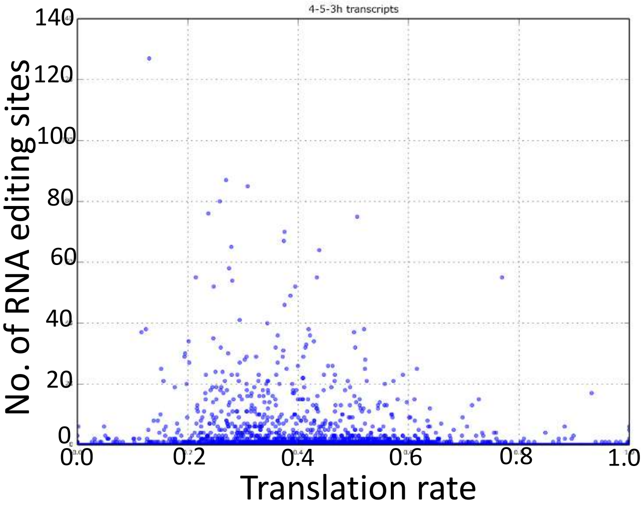

5.3hpf

Pearson: r=-0.071 p=3.09e-18

Spearman: r=-0.105 p=2.68e-38

Supplementary Figure 2. Translation rates and number of editing sites for 1.5 hpf and 5.3 hpf transcripts. Translation rates are expressed as ratio of polysome bound to sum of bound and unbound fractions of a given transcript [54]. Thus, translation rate of 1.0 means all expressed transcript molecules are associated with polysome, while 0.0 means none of expressed transcript molecules are associated with polysome. Source data is provided in source data file.

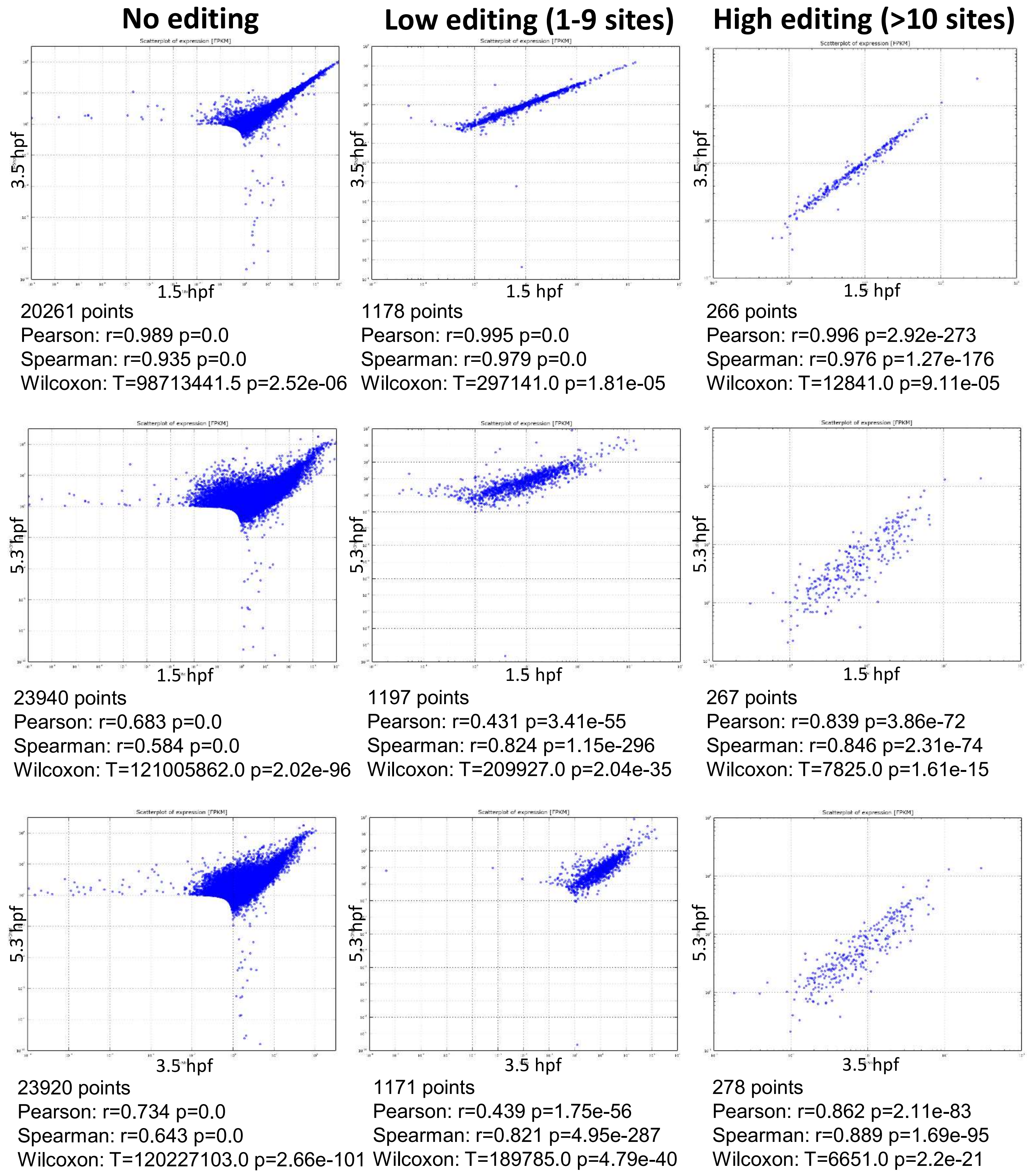

Supplementary Figure 3. Comparison of expression levels between two developmental stages of non-edited, low-edited, and highly edited transcripts. Two-sided statistical test was applied. Source data is provided in source data file.

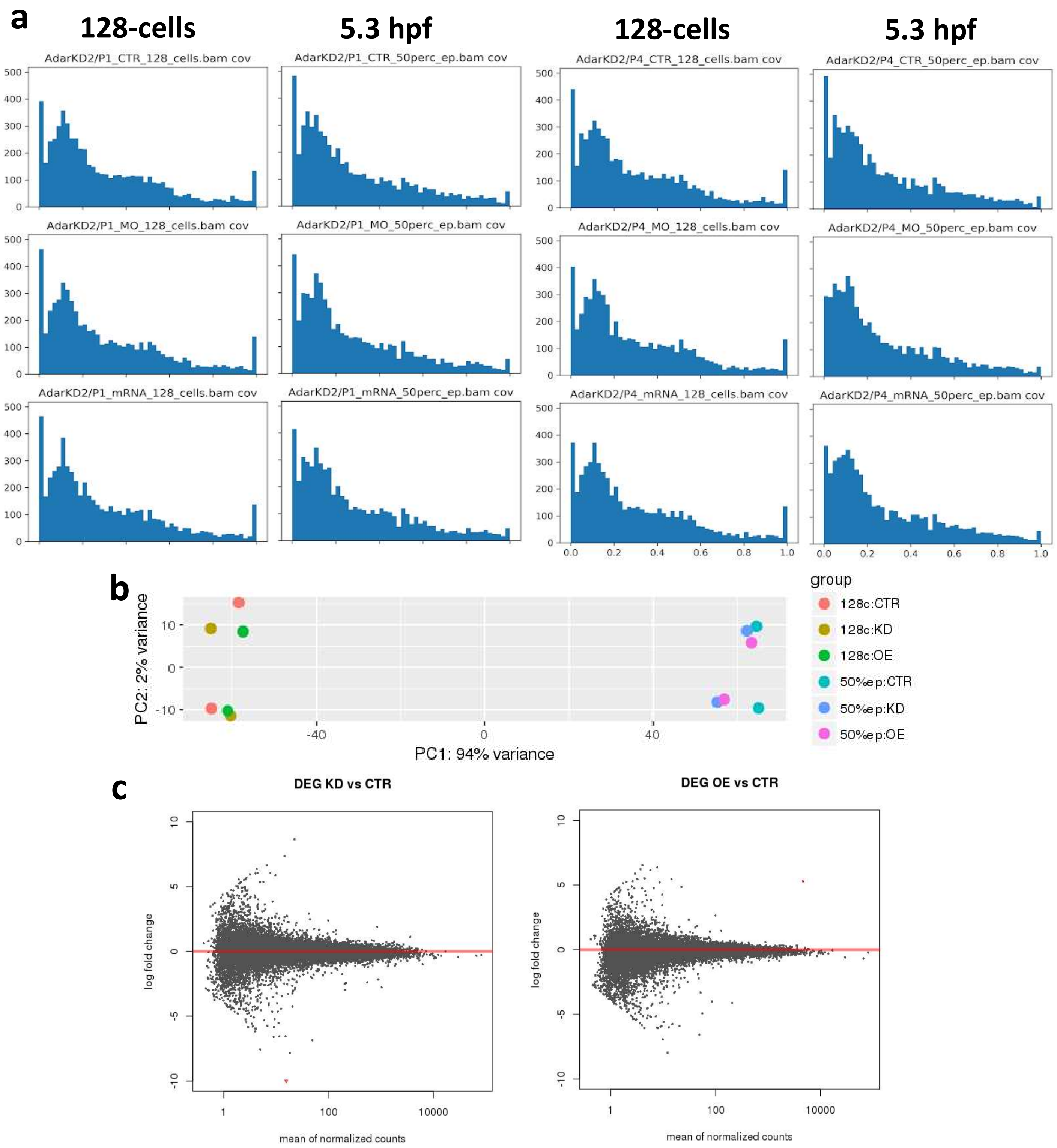

Supplementary Figure 4. Global RNA editing and gene expression profile of Adar knockdown and overexpression. (A) Penetrance of RNA editing expressed as number of transcripts vs. fraction of editing within a transcript species in 128-cell (left column) and 5.3 hpf (right column). (B) Principal component analysis of control, Adar knockdown and overexpression samples based on their transcriptome profile. (C) Differential expression analysis of Adar knockdown and overexpression vs. control. No genes were differentially expressed at $p < 0.05$. Sequencing read counts were normalized with DESeq2 standard method. Statistical significance is determined by the Wald test corrected for multiple testing using the Benjamini and Hochberg method using DESeq2. Source data is provided in source data file.

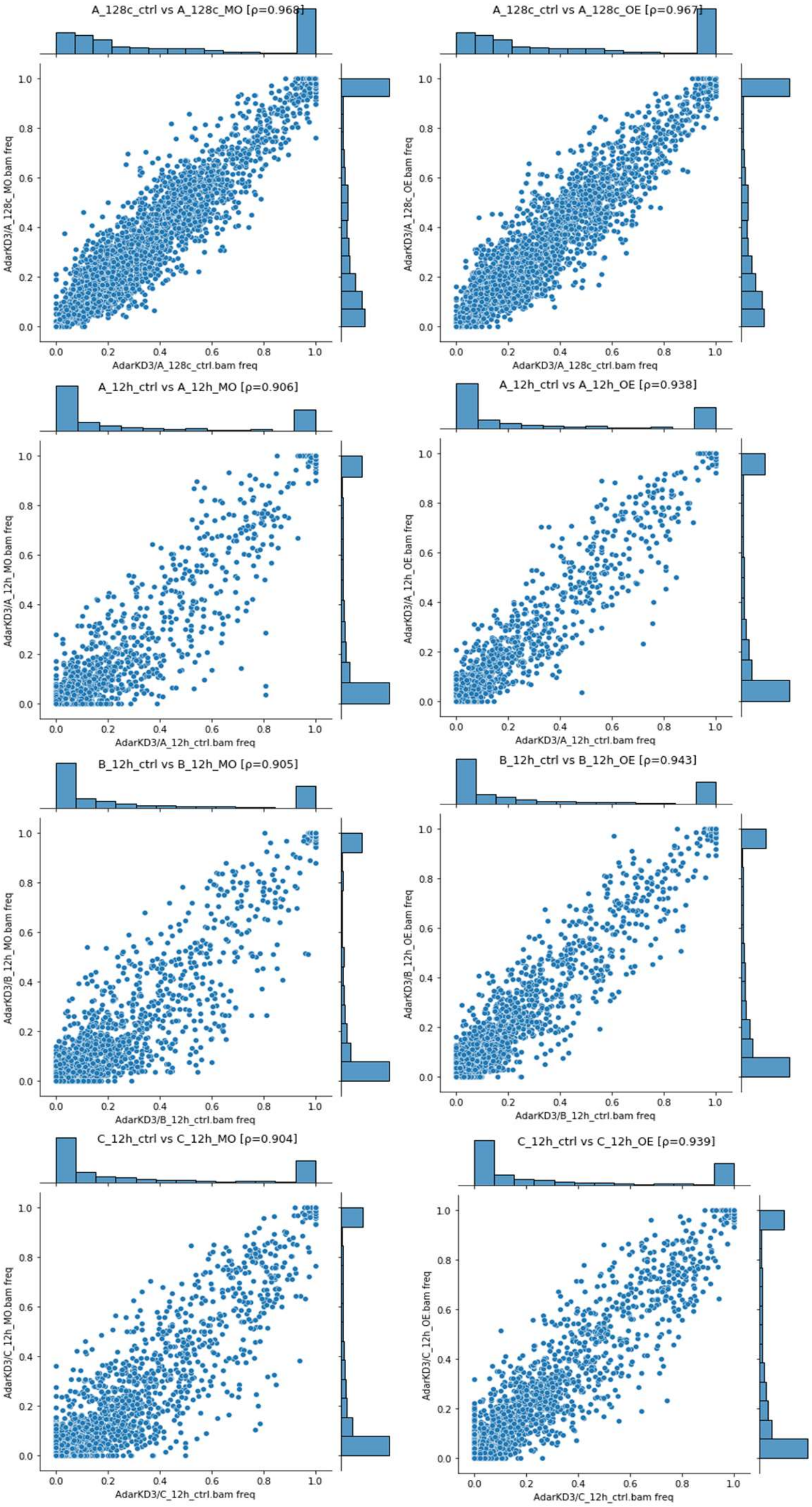

Supplementary Figure 5. Comparison of RNA editing frequency between control and Adar KD or OE. RNA editing frequencies for each transcript is plotted for each sample in 128-cell and 12 hpf stages. Spearman's rank correlation coefficient (ρ) is given in every figure title. Source data is provided in source data file.

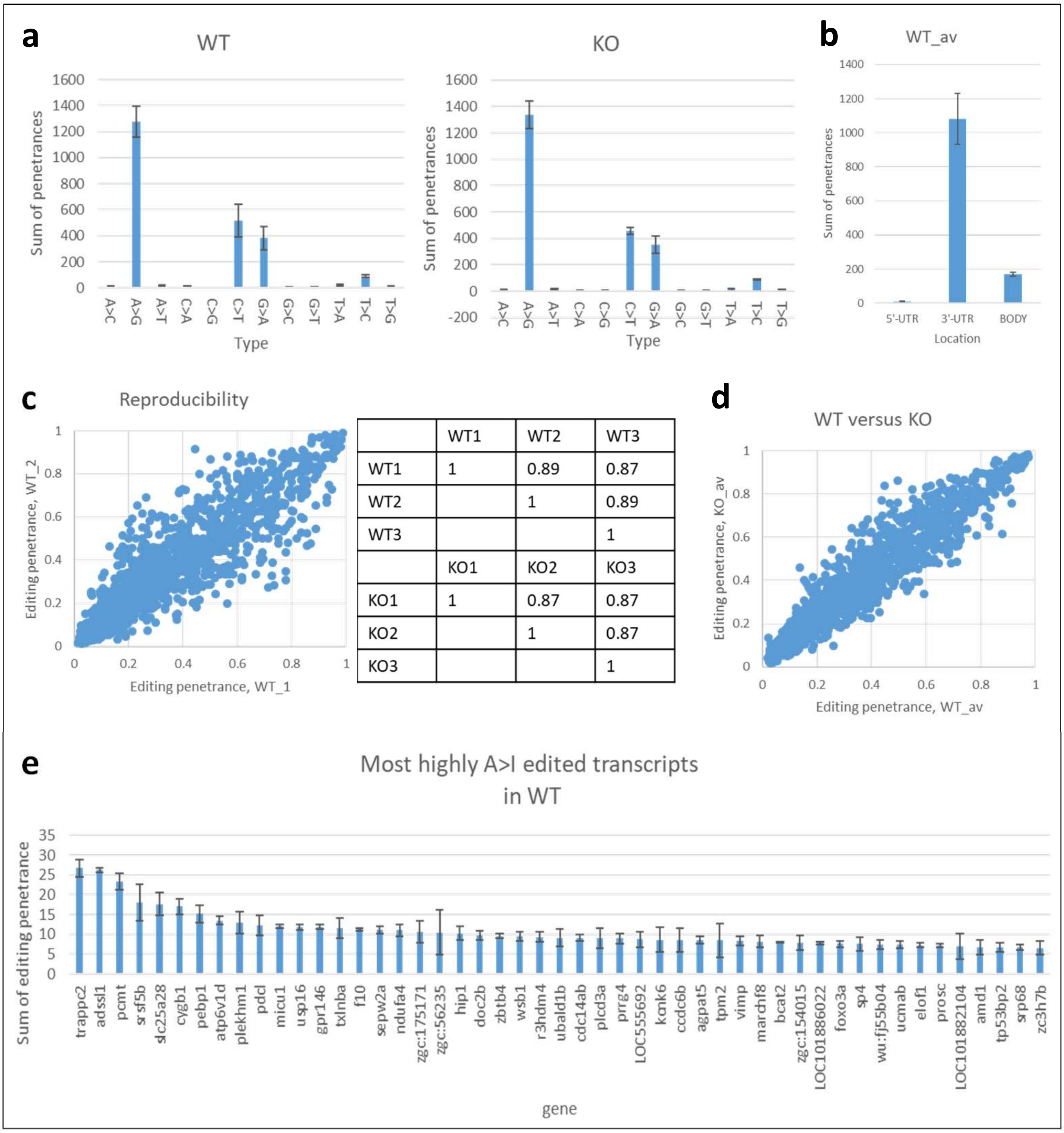

Supplementary Figure 6. RNA editing profile at 7 dpf in wild-type and *adar-/-* mutants. (A) types of base conversions observed in wild-type and *adar-/-* mutants (KO). Note the prevalence of A>G conversions similar to that observed during embryonic stages. (B) Distribution of editing sites along the transcript features. Similar to that observed in embryonic stages, editing events predominantly occur at the 3'-UTR of transcripts. (C) Reproducibility between samples (left) and the Pearson's correlation coefficient between samples, (D) Correlation of editing profiles (based on editing sites) between wild-type and KO (ρ = 0.94). (E) The most highly edited genes at 7 dpf. Data (A, B, E) are presented as mean values +/- SD (n=3). Source data: Source data file, Supplementary Data 12.
