# Supplementary material for: Adar-mediated A-to-I editing is required for embryonic patterning and innate immune response regulation in zebrafish
Source: Nat Commun. 2022 Sep 20;13:5520. doi: 10.1038/s41467-022-33260-6 (PMC9489775; doi:10.1038/s41467-022-33260-6)
Supplement: Supplementary file 2 — Description of Additional Supplementary Files [file 41467_2022_33260_MOESM2_ESM.pdf]

Description of Additional Supplementary Files

File Name: Supplementary Data 1
Description: RNA editing statistics at 1.5 hpf, 3.5 hpf, and 5.3 hpf stages.

File Name: Supplementary Data 2
Description: RNA editing frequency in various classes of repeat elements.

File Name: Supplementary Data 3
Description: RNA editing penetrance showing the number of editing sites in a single read relative to the total editing events in a given transcript region.

File Name: Supplementary Data 4
Description: Genes containing at least 2 editing sites.

File Name: Supplementary Data 5
Description: Enriched GO terms among genes containing at least 2 editing sites. Functional enrichment analysis was performed using DAVID which employs Fisher's Exact test with Benjamini, Bonferroni corrections.

File Name: Supplementary Data 6
Description: Editing frequencies in Adar knockdown and overexpression conditions at 128-cell and 5.3 hpf stages.

File Name: Supplementary Data 7
Description: Differentially expressed genes in Adar knockdown and overexpression at 12 hpf. Statistical significance is determined by the Wald test corrected for multiple testing using the Benjamini and Hochberg method using DESeq2.

File Name: Supplementary Data 8
Description: Enriched GO terms among differentially expressed genes at 12 hpf. Functional enrichment analysis was performed using DAVID which employs Fisher's Exact test with Benjamini, Bonferroni corrections.

File Name: Supplementary Data 9
Description: List of primers used in this study.

File Name: Supplementary Data 10
Description: Mapping statistics of sequencing data.

File Name: Supplementary Data 11
Description: Differentially expressed genes in Adar homozygous mutants at 7 dpf. Statistical significance is determined by the Wald test corrected for multiple testing using the Benjamini and Hochberg method using DESeq2.

File Name: Supplementary Data 12
Description: RNA editing statistics at 7 dpf.
