# Supplementary material for: Adar-mediated A-to-I editing is required for embryonic patterning and innate immune response regulation in zebrafish
Source: Nat Commun. 2022 Sep 20;13:5520. doi: 10.1038/s41467-022-33260-6 (PMC9489775; doi:10.1038/s41467-022-33260-6)
Supplement: Supplementary file 4 — Reporting Summary [file 41467_2022_33260_MOESM4_ESM.pdf]

nature portfolio

Corresponding author(s): Cecilia Winata

Last updated by author(s): Aug 8, 2022

Reporting Summary

Nature Portfolio wishes to improve the reproducibility of the work that we publish. This form provides structure for consistency and transparency in reporting. For further information on Nature Portfolio policies, see our Editorial Policies and the Editorial Policy Checklist.

Statistics

For all statistical analyses, confirm that the following items are present in the figure legend, table legend, main text, or Methods section.

n/a	Confirmed	
☐	☒	The exact sample size (*n*) for each experimental group/condition, given as a discrete number and unit of measurement
☐	☒	A statement on whether measurements were taken from distinct samples or whether the same sample was measured repeatedly
☐	☒	The statistical test(s) used AND whether they are one- or two-sided<br>*Only common tests should be described solely by name; describe more complex techniques in the Methods section.*
☐	☒	A description of all covariates tested
☐	☒	A description of any assumptions or corrections, such as tests of normality and adjustment for multiple comparisons
☐	☒	A full description of the statistical parameters including central tendency (e.g. means) or other basic estimates (e.g. regression coefficient) AND variation (e.g. standard deviation) or associated estimates of uncertainty (e.g. confidence intervals)
☐	☒	For null hypothesis testing, the test statistic (e.g. *F*, *t*, *r*) with confidence intervals, effect sizes, degrees of freedom and *P* value noted<br>*Give P values as exact values whenever suitable.*
☒	☐	For Bayesian analysis, information on the choice of priors and Markov chain Monte Carlo settings
☒	☐	For hierarchical and complex designs, identification of the appropriate level for tests and full reporting of outcomes
☐	☒	Estimates of effect sizes (e.g. Cohen's *d*, Pearson's *r*), indicating how they were calculated

Our web collection on statistics for biologists contains articles on many of the points above.

Software and code

Policy information about availability of computer code

Data collection	no software was used
Data analysis	Reads were aligned to the zebrafish genome assembly GRCz10 using STAR v2.7.7a [68] and samtools v1.11 [69]. Expression quantification was performed using HTSeq v0.11.2 [70]. Differential expression was performed using DESeq2 (R v3.6.3) [71]. Multiple testing was done by applying the Benjamini-Hochberg correction as implemented in DESeq2 with adjusted p-values <0.05 called as statistically significant. For the Adar zygotic function analysis, sequencing data were demultiplexed, and preprocessed with a Snakemake pipeline. Reads were subjected to quality-control using fastp [72]. At the 5′- and 3′-ends, 15 and 3 nucleotides were removed respectively based on atypical base composition according to fastqc/multiqc [73]. The truncated reads were then aligned to the zebrafish genome assembly GRCz11 using STAR v2.7.7a [68], paired (option fixmate), sorted, and (again) deduplicated (option markdup -r) using samtools [69]. Based on the zebrafish genome annotation (z11.105). the reads were then translated to feature counts using HTSeq (option -s reverse) [70]. From the feature counts onwards, analysis was done in R using custom scripts. Differential expression analysis was carried out in DESeq2 [71]. Significantly overexpressed and underexpressed genes (-2 > log2FC > 2; padj < 0.05) were annotated using bioMart [74], and subjected to GO analysis using clusterProfiler [75]. The analysis is available under https://github.com/mbochtler/zebrafish_ADAR/7dpf/analysis_overexpression.<br>Putative RNA editing sites based on DNA- and RNA-seq input were detected using REDiscover (https://github.com/lpryszcz/REDiscover). The script automatically eliminates low quality and duplicate reads. It utilizes samtools mpileup to generate a text file from input bam files. Options -q 15 -Q 20, were used, i.e. a minimal mapping quality of 15 and a minimal base call quality of 20 were required.

For manuscripts utilizing custom algorithms or software that are central to the research but not yet described in published literature, software must be made available to editors and reviewers. We strongly encourage code deposition in a community repository (e.g. GitHub). See the Nature Portfolio guidelines for submitting code & software for further information.

Data

Policy information about availability of data

All manuscripts must include a data availability statement. This statement should provide the following information, where applicable:
- Accession codes, unique identifiers, or web links for publicly available datasets
- A description of any restrictions on data availability
- For clinical datasets or third party data, please ensure that the statement adheres to our policy

All sequencing data have been deposited in the GEO database under accession number GSE182714. Other public datasets used: SNP data for zebrafish Zv9 (https://snpfisher.nichd.nih.gov/snpfisher/tracks.html), GRCz10, GRCz11.

Human research participants

Policy information about studies involving human research participants and Sex and Gender in Research.

Reporting on sex and gender	no human participants were used in this study
Population characteristics	no human participants were used in this study
Recruitment	no human participants were used in this study
Ethics oversight	no human participants were used in this study

Note that full information on the approval of the study protocol must also be provided in the manuscript.

Field-specific reporting

Please select the one below that is the best fit for your research. If you are not sure, read the appropriate sections before making your selection.

☒ Life sciences ☐ Behavioural & social sciences ☐ Ecological, evolutionary & environmental sciences

For a reference copy of the document with all sections, see nature.com/documents/nr-reporting-summary-flat.pdf

Life sciences study design

All studies must disclose on these points even when the disclosure is negative.

Sample size	No statistical method was used to predetermine sample size. Group size was based on our previous experience. RNA editing discovery was performed with a single trio of parental genomic sequencing and offspring transcriptomes at three different time points. The reliability of the initial A-to-I editing discovery is dependent on the parental genomic sequence. Therefore, sequencing was performed in a single trio of samples (male and female parental genome and offspring transcriptome) as the cost of sequencing additional sets are prohibitive. The offspring transcriptome was obtained as a result of pooling 20 individuals which therefore dilutes out any biological variance. To assess the effect of Adar KD and OE, two replicates of 20 pooled embryos from the first two time points and were isolated for RNA extraction. Three replicates for the 12 hpf time point analysis were collected in a second round of experiments, from offspring of a separate mating pair. Analysis of adar zygotic function were performed at 7 dpf from three replicates of 3 individuals each. The number of replicates were sufficient to allow correlation analysis and/or statistical testing. High correlation between replicates were obtained in all cases which supported the soundness of our observations. Whole-mount in situ hybridization was performed on 24 hpf wild-type, adar morpholino-injected and adar mRNA-injected embryos. Results were obtained from at least 3 different experiments on embryos from random pairs. Genetic compensation analysis was performed on 5 individuals from each WT and adar homozygous mutant groups.
Data exclusions	No data was excluded from the analysis
Replication	Unless otherwise stated, all experiments included biological replicates and were analyzed using the same bioinformatics pipelines, wherever possible keeping analyses parameters constant to ensure reproducibility. All attempts at replication were successful. RNA editing discovery was performed with a single trio of parental genomic sequencing and offspring transcriptomes at three different time points. The reliability of the initial A-to-I editing discovery is dependent on the parental genomic sequence. Therefore, sequencing was performed in a single trio of samples (male and female parental genome and offspring transcriptome) as the cost of sequencing additional sets are prohibitive. The offspring transcriptome was obtained as a result of pooling multiple embryos (20 individuals) which therefore dilutes out any biological variance.
Randomization	Samples were allocated to experimental groups according to their biological attributes which include developmental stage, genotype, or treatment type. These attributes defines each group clearly enough such that randomization is not necessary. Moreover, randomization is not relevant as the analyses of compared groups were performed with the same pipelines and parameters and thus not subject to observer bias.
Blinding	Investigators were not blinded to group allocation during data collection. Blinding is not relevant as the analyses of compared groups were

Blinding | performed with the same pipelines and parameters and thus not subject to observer bias.

Reporting for specific materials, systems and methods

We require information from authors about some types of materials, experimental systems and methods used in many studies. Here, indicate whether each material, system or method listed is relevant to your study. If you are not sure if a list item applies to your research, read the appropriate section before selecting a response.

Materials & experimental systems

n/a	Involved in the study
☐	☒ Antibodies
☒	☐ Eukaryotic cell lines
☒	☐ Palaeontology and archaeology
☐	☒ Animals and other organisms
☒	☐ Clinical data
☒	☐ Dual use research of concern

Methods

n/a	Involved in the study
☒	☐ ChIP-seq
☒	☐ Flow cytometry
☒	☐ MRI-based neuroimaging

Antibodies

Antibodies used: anti-DIG-AP antibody (Roche Cat. No. 11093274910), lot number: 14608125

Validation: This antibody is commonly used for whole mount in situ hybridization and has been reported in multiple publications as summarized in the manufacturer's website (https://www.sigmaaldrich.com/PL/pl/product/roche/11093274910?gclid=CjwKCAjwlqOXBhBqEiwA-hhitJyxmn1PJtbtQ9bBmz6bt1iX2p6T05Yspsyg2E-STigdAsRX_dqrURoCtd4QAvD_BwE).

Animals and other research organisms

Policy information about studies involving animals; ARRIVE guidelines recommended for reporting animal research, and Sex and Gender in Research

Laboratory animals: Zebrafish adar mutant line (on the background of AB strain wild-type) was maintained and propagated as heterozygous carriers which are viable and exhibit no phenotype. adar-/- homozygous individuals were obtained from incross of heterozygous carriers and were harvested for RNA isolation at 7 dpf. All other experiments were performed in embryos up to 5 dpf which do not fall into the regulatory frameworks of animal experimentation according to EU Directive 2010/63/EU on the protection of animals used for scientific purposes.

Wild animals: the study did not involve wild animals.

Reporting on sex: sex was not considered as the analyses were done in a pool of emrbyos. In addition, zebrafish has not developed any sexual characteristics nor reached sexual maturity by the latest time analyses were performed.

Field-collected samples: the study did not involve samples collected from the field.

Ethics oversight: The study follows the guidelines on animal experimentation according to the EU Directive 2010/63/EU on the protection of animals used for scientific purposes.

Note that full information on the approval of the study protocol must also be provided in the manuscript.
